# Supplementary material for: Evaluating the clinical utility of large language models for hepatocellular carcinoma treatment recommendations: A nationwide retrospective registry study
Source: PLoS Med. 2026 Jan 13;23(1):e1004855. doi: 10.1371/journal.pmed.1004855 (PMC12799000; doi:10.1371/journal.pmed.1004855)
Supplement: S3 Fig — (DOCX) [file pmed.1004855.s003.docx]

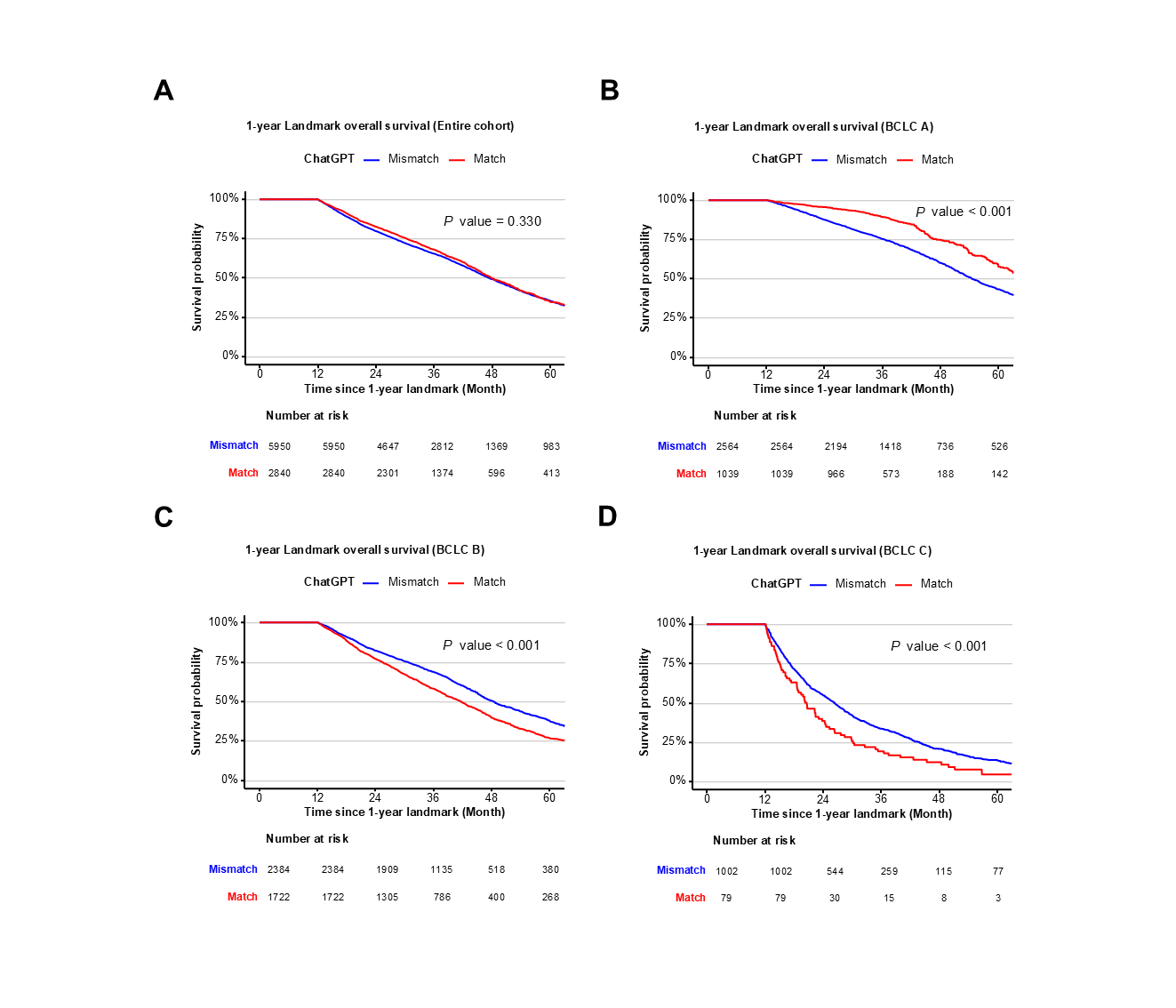


**S3 Fig. 1-year landmark overall survival according to concordance between ChatGPT 4o-recommended and physician-administered treatments in patients with HCC.** Kaplan–Meier survival curves depict overall survival from the 1-year landmark (patients surviving ≥ 12 months after diagnosis) comparing those whose actual treatment matched ChatGPT’s recommendation (red line) versus those whose treatment differed (blue line). (A) Entire cohort; (B) BCLC stage A; (C) BCLC stage B; (D) BCLC stage C. *P* values were calculated using the log-rank test.
