# Supplementary material for: Evaluating the clinical utility of large language models for hepatocellular carcinoma treatment recommendations: A nationwide retrospective registry study
Source: PLoS Med. 2026 Jan 13;23(1):e1004855. doi: 10.1371/journal.pmed.1004855 (PMC12799000; doi:10.1371/journal.pmed.1004855)
Supplement: S6 Fig — (DOCX) [file pmed.1004855.s006.docx]

**
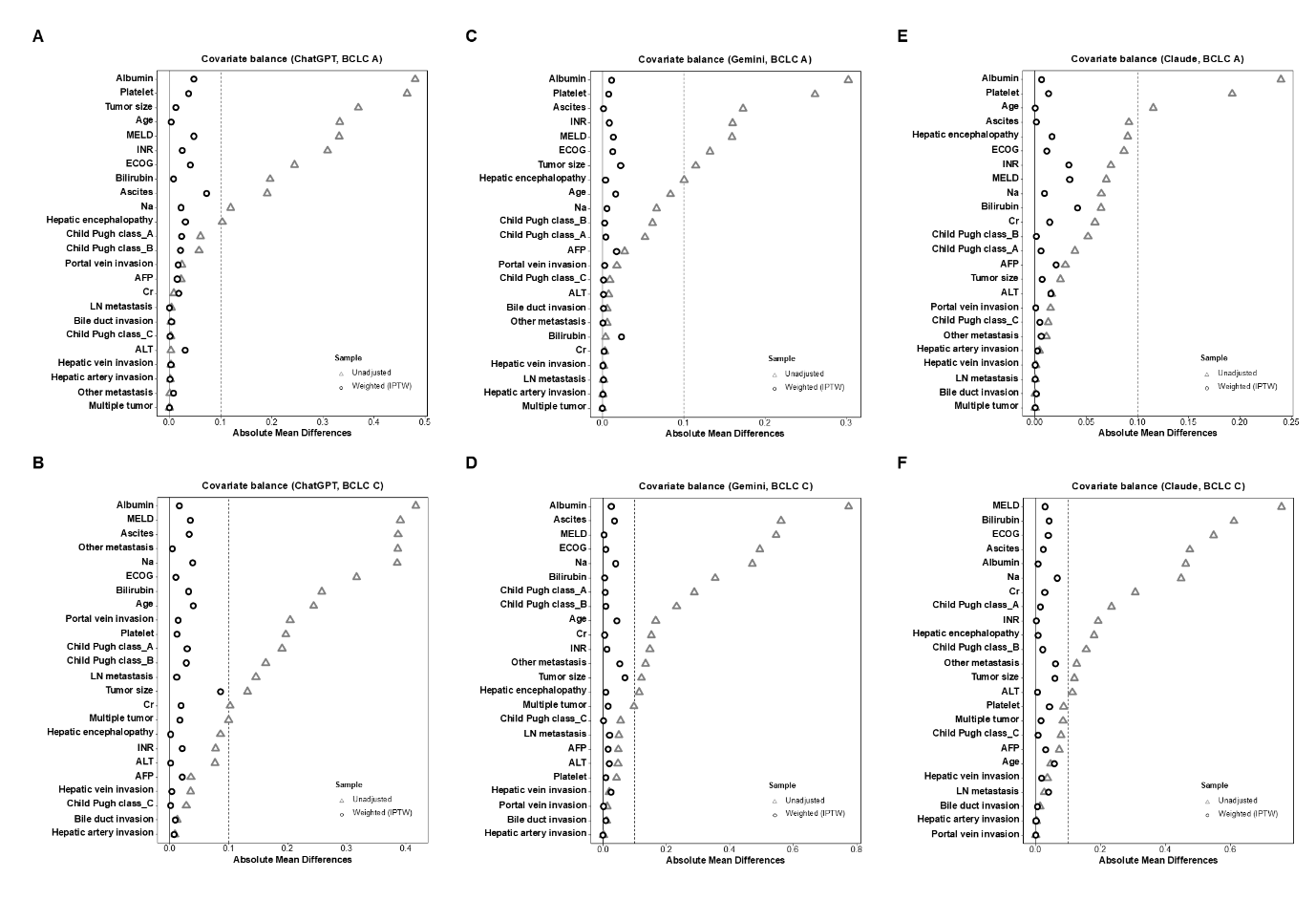
S6 Fig. Covariate balance before and after weighting by LLM and BCLC stage.** Panels A–F show absolute standardized mean differences (SMDs) of baseline covariates before and after inverse probability weighting (IPTW) among patients with BCLC stage A (A, C, E) and stage C (B, D, F), according to the large language model (LLM) generating treatment recommendations (ChatGPT 4o, Gemini 2.0, Claude 3.5). Triangles represent weighted (IPTW) samples, and circles represent unadjusted samples. The vertical dashed line denotes the conventional threshold of SMD < 0.1, indicating adequate covariate balance between the LLM-matched and mismatched groups. ECOG, Eastern Cooperative Oncology Group; ALT, alanine aminotransferase; AFP, alpha-fetoprotein; MELD, Model for End-Stage Liver Disease; LN, lymph node.
