# Supplementary material for: Evaluating the clinical utility of large language models for hepatocellular carcinoma treatment recommendations: A nationwide retrospective registry study
Source: PLoS Med. 2026 Jan 13;23(1):e1004855. doi: 10.1371/journal.pmed.1004855 (PMC12799000; doi:10.1371/journal.pmed.1004855)
Supplement: S7 Fig — (DOCX) [file pmed.1004855.s007.docx]

**
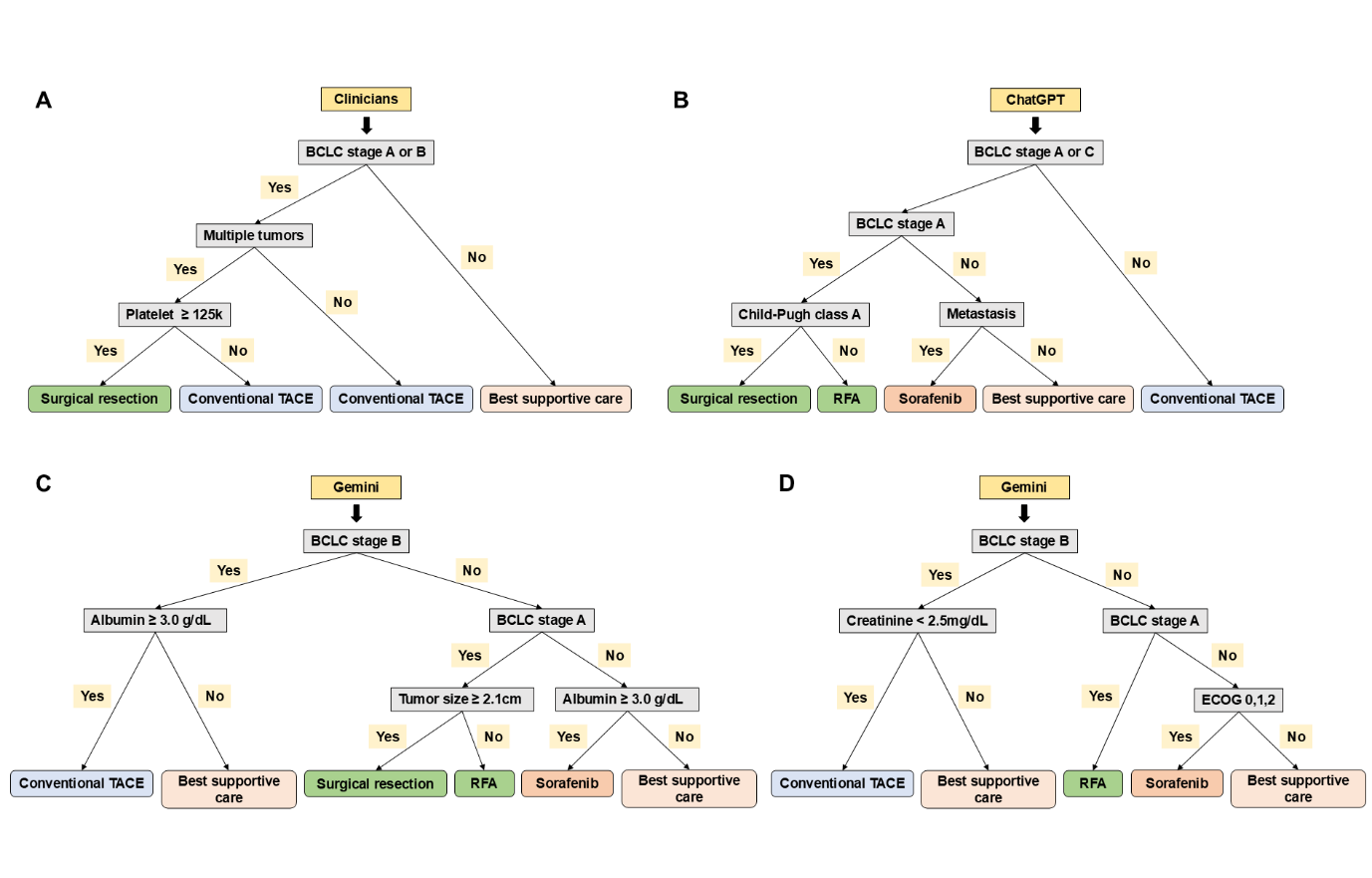
**

**S7 Fig. Simplified decision trees for treatment recommendations by clinicians and LLMs.** (A) A simplified decision tree model trained on physician decisions. (B–D) Decision tree classifiers built using ChatGPT 4o (B), Gemini 2.0 (C), and Claude 3.5 (D) recommendations.
