# Supplementary material for: Evaluating the clinical utility of large language models for hepatocellular carcinoma treatment recommendations: A nationwide retrospective registry study
Source: PLoS Med. 2026 Jan 13;23(1):e1004855. doi: 10.1371/journal.pmed.1004855 (PMC12799000; doi:10.1371/journal.pmed.1004855)
Supplement: S8 Fig — (DOCX) [file pmed.1004855.s008.docx]

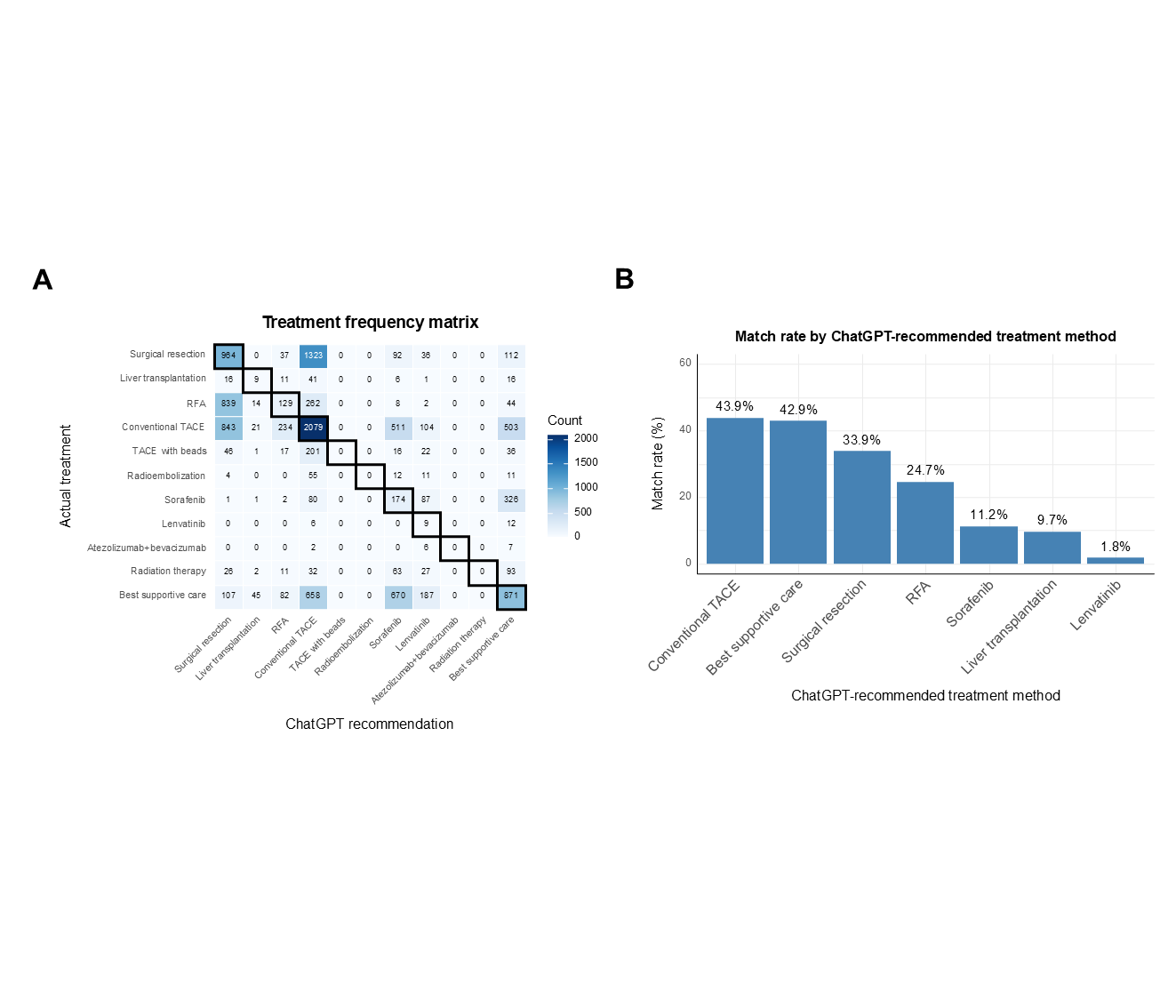


**S8 Fig. Concordance between ChatGPT 4o-recommended treatments and actual clinical decisions in HCC patients.** (A) A treatment frequency matrix comparing ChatGPT-recommended treatment options (x-axis) with the actual treatments administered by physicians (y-axis). Diagonal elements represent matched cases, with higher counts indicating concordance. (B) Match rates (%) between ChatGPT recommendations and actual treatments for each recommended method. Only treatment categories with ≥ 5 cases were included for clarity.
