# Supplementary material for: Evaluating the clinical utility of large language models for hepatocellular carcinoma treatment recommendations: A nationwide retrospective registry study
Source: PLoS Med. 2026 Jan 13;23(1):e1004855. doi: 10.1371/journal.pmed.1004855 (PMC12799000; doi:10.1371/journal.pmed.1004855)
Supplement: S11 Fig — (DOCX) [file pmed.1004855.s011.docx]

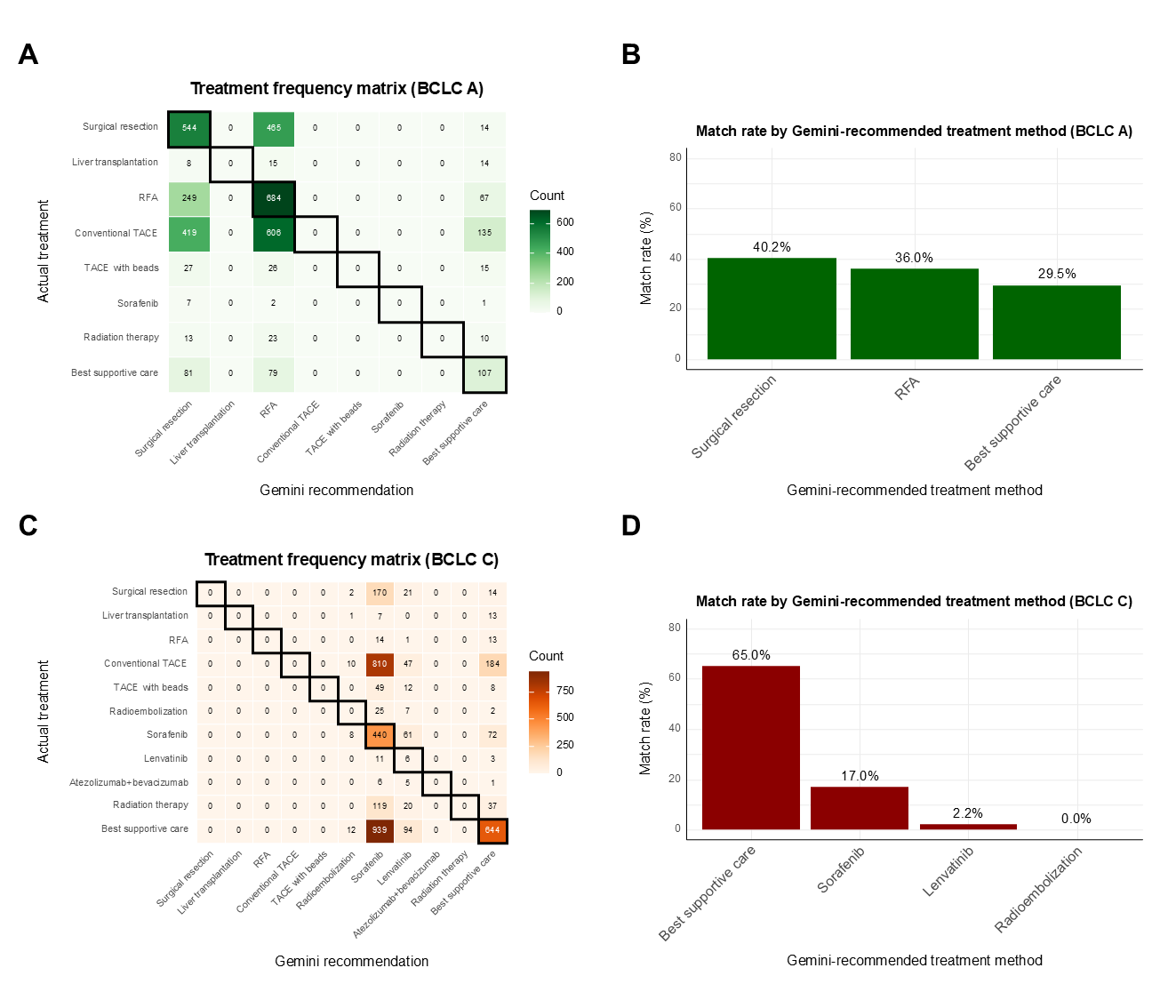


**S11 Fig. Subgroup concordance analysis between Gemini 2.0-based treatment suggestions and clinical practice across BCLC stages.** (A) Matrix visualization of treatment frequencies comparing Gemini-recommended options (x-axis) with actual therapies provided by clinicians (y-axis) in patients with BCLC stage A. Cells on the diagonal denote concordant decisions, with darker shades indicating higher agreement. (B) Bar plot summarizing the match rate (%) for each treatment recommended by Gemini among patients with BCLC stage A. (C) Frequency matrix for BCLC stage C patients, showing alignment between Gemini's suggestions and real-world physician decisions. (D) Match rates for each Gemini recommendation in BCLC stage C patients. To ensure interpretability, treatment categories with fewer than five cases were excluded from the bar plots.
