# Supplementary material for: Evaluating the clinical utility of large language models for hepatocellular carcinoma treatment recommendations: A nationwide retrospective registry study
Source: PLoS Med. 2026 Jan 13;23(1):e1004855. doi: 10.1371/journal.pmed.1004855 (PMC12799000; doi:10.1371/journal.pmed.1004855)
Supplement: S13 Fig — (DOCX) [file pmed.1004855.s013.docx]

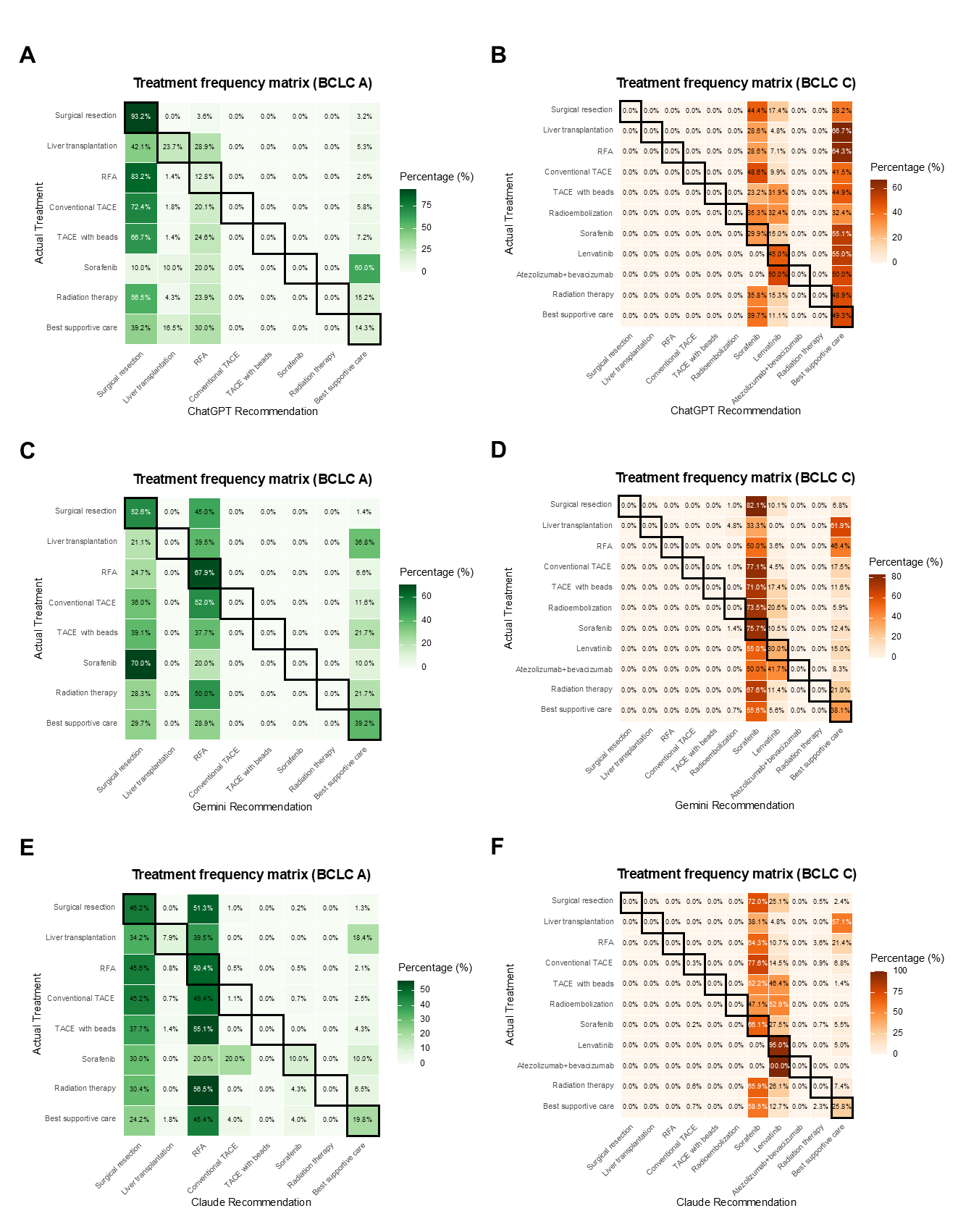


**S13 Fig. Subgroup concordance analysis between LLM-based treatment suggestions and clinical practice across BCLC stages.** (A–B) Matrix visualization of treatment frequencies comparing ChatGPT 4o-recommended options (x-axis) with actual therapies administered by clinicians (y-axis) in patients with BCLC stage A (A) and stage C (B). (C–D) Corresponding matrices for Gemini 2.0-based recommendations in BCLC stage A (C) and stage C (D). (E–F) Equivalent matrices for Claude 3.5-based recommendations in BCLC stage A (E) and stage C (F). Each cell denotes the percentage of patients for whom the model-recommended therapy (x-axis) corresponded to the actual treatment administered (y-axis), with darker shades indicating higher frequencies.
