# Supplementary material for: Evaluating the clinical utility of large language models for hepatocellular carcinoma treatment recommendations: A nationwide retrospective registry study
Source: PLoS Med. 2026 Jan 13;23(1):e1004855. doi: 10.1371/journal.pmed.1004855 (PMC12799000; doi:10.1371/journal.pmed.1004855)
Supplement: S14 Fig — (DOCX) [file pmed.1004855.s014.docx]

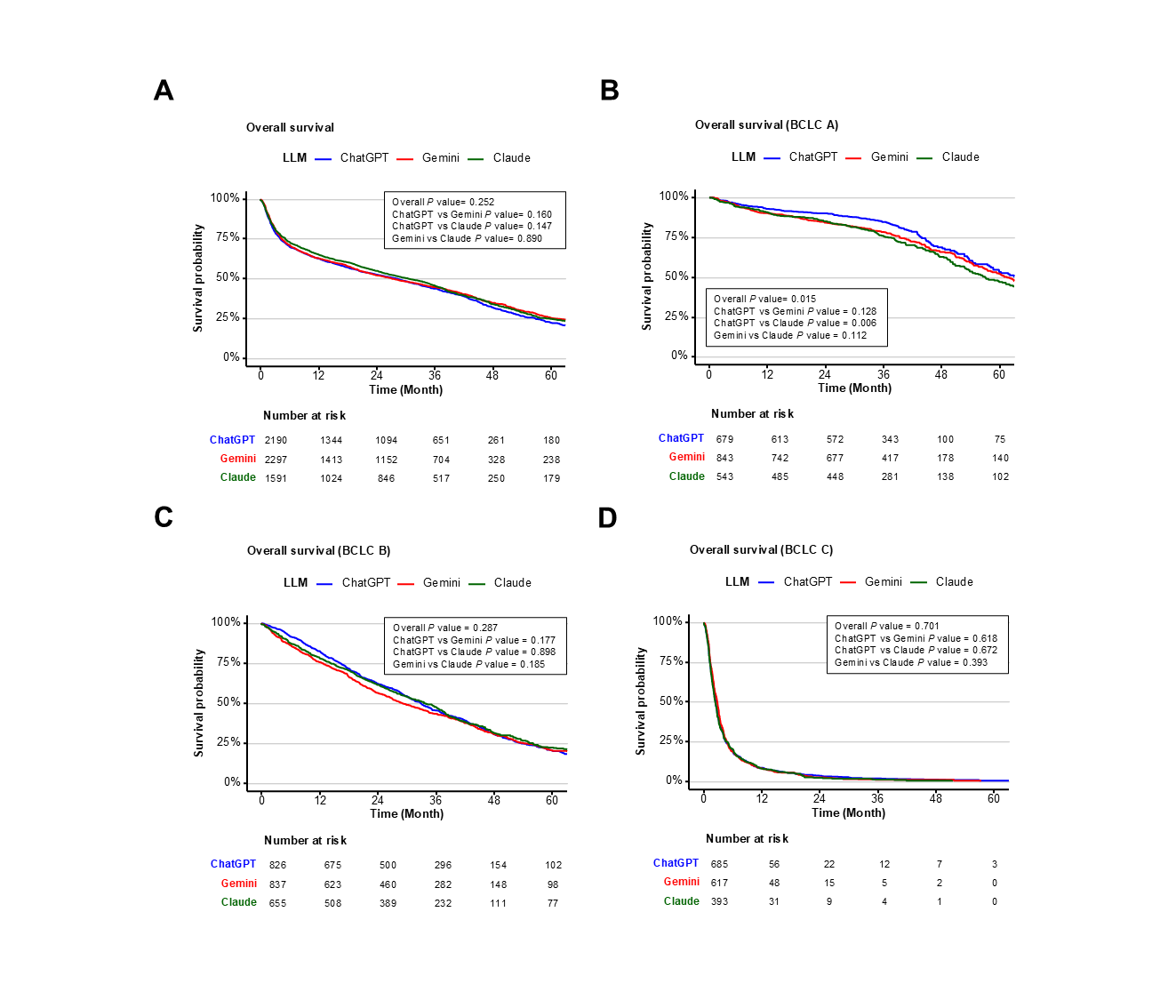


**S14 Fig. Kaplan–Meier curves of overall survival according to the large language models (ChatGPT 4o, Gemini 2.0, and Claude 3.5) before IPTW.** (A) Entire cohort. (B) BCLC stage A. (C) BCLC stage B. (D) BCLC stage C. *P* values for overall and pairwise comparisons are shown in each panel.
