# Supplementary material for: Evaluating the clinical utility of large language models for hepatocellular carcinoma treatment recommendations: A nationwide retrospective registry study
Source: PLoS Med. 2026 Jan 13;23(1):e1004855. doi: 10.1371/journal.pmed.1004855 (PMC12799000; doi:10.1371/journal.pmed.1004855)
Supplement: S1 Table — (DOCX) [file pmed.1004855.s015.docx]

**S1 Table. CHART checklist.**

| **Section** | **Item** | **Topic** | **Checklist item** | **Page** |
| --- | --- | --- | --- | --- |
| **Title & Abstract** | **1a** | **Title** | State that the study is assessing one or more generative AI-driven chatbots for clinical evidence or health advice. | 3 |
|  | **1b** | **Abstract/Summary** | Apply a structured format, if applicable. | 3 |
| **Introduction** | **2a** | **Background** | State the scientific background, rationale, and healthcare context for evaluating the generative AI-driven chatbot(s), referencing relevant literature when applicable. | 6-7 |
|  | **2b** | **Aims & Research Questions** | State the aims and research questions including the target audience, intervention, comparator(s), and outcome(s). | 6-7 |
| **Methods** | **3a** | **Model Identifiers** | State the name and version identifier(s) of the generative AI model(s) and chatbot(s) under evaluation, as well as their date of release or last update. | 8-9 |
|  | **3b** | **Model Identifiers** | State whether the generative AI model(s) and chatbot(s) are open-source or closed-source/proprietary. | 8-9 |
|  | **4a** | **Model Details** | State whether the generative AI model was a base model or a novel base model, tuned model, or fine-tuned model. | 8-9 |
|  | **4b** | **Model Details** | If a base model is used, cite its development in sufficient detail to identify the model. | 8-9 |
|  | **4c** | **Model Details** | If a novel base model, tuned model, or fine-tuned model is used, describe the pre- and/or postimplementation/deployment data and parameters. | 8-9 |
|  | **5a** | **Prompt Engineering** | Describe the evolution of study prompt development. | 8-9 |
|  | **5ai** | **Prompt Engineering** | Describe the sources of prompts. | 8-9 |
|  | **5aii** | **Prompt Engineering** | State the number and characteristics of the individual(s) involved in prompt engineering. | 8-9 |
|  | **5aiii** | **Prompt Engineering** | Provide details of any patient and public involvement during prompt engineering. | 8-9 |
|  | **5b** | **Prompt Engineering** | Provide study prompts. | 8-9 |
|  | **6a** | **Query Strategy** | State route of access to generative AI model. | 8-9 |
|  | **6b** | **Query Strategy** | State the date(s) and location(s) of queries for the generative AI-driven chatbot(s) including the day, month, and year as well as city and country. | 8-9 |
|  | **6c** | **Query Strategy** | Describe whether prompts were input into separate chat session(s). | 8-9 |
|  | **6d** | **Query Strategy** | Provide all generative AI-driven chatbot output/responses | 8-9 |
|  | **7a** | **Performance Evaluation** | Define the ground truth or reference standard used to define successful generative AI-driven chatbot performance. | 8-9 |
|  | **7b** | **Performance Evaluation** | Describe the process undertaken for generative AI-driven chatbot performance evaluation. | 8-9 |
|  | **7bi** | **Performance Evaluation** | State the number and characteristics of team members involved in performance evaluation. | 8-9 |
|  | **7bii** | **Performance Evaluation** | Provide details of any patients and public involvement during the evaluation process. | 8-9 |
|  | **7biii** | **Performance Evaluation** | State whether evaluators were blinded to the identity of the generative AI-driven chatbot(s) under assessment. | 8-9 |
|  | **8** | **Sample Size** | Report how the sample size was determined. | 8-9 |
|  | **9a** | **Data Analysis** | Describe statistical analysis methods, including any evaluation of reproducibility of generative AI-driven chatbot responses. | 8-9 |
|  | **9ai** | **Data Analysis** | Report the measures used for performance evaluation. | 8-9 |
| **Results** | **10a** | **Results** | Report the alignment between generative AI-driven chatbot output and ground truth or reference standard using quantitative or mixed methods approaches as applicable. | 11-18 |
|  | **10b** | **Results** | For responses deviating from the ground truth or reference standard, state the nature of the difference(s). | 11-18 |
|  | **10c** | **Results** | Report the assessment for potentially harmful, biased, or misleading responses. | 11-18 |
| **Discussion** | **11a** | **Discussion** | Interpret study findings in the context of relevant evidence. | 19-20 |
|  | **11b** | **Discussion** | Describe the strengths and limitations of the study. | 21 |
|  | **11c** | **Discussion** | Describe the potential implications for practice, education, policy, regulation, and research. | 19-22 |
| **Open Science** | **12a** | **Disclosures** | Report any relevant conflicts of interest for all authors. | 1 |
|  | **12b** | **Funding** | Report sources of funding and their role in the conduct and reporting of the study. | 1 |
|  | **12c** | **Ethics** | Describe the process undertaken for ethical approval. | 2 |
|  | **12ci** | **Ethics** | Describe the measures taken to safeguard data privacy of patient health information, as applicable. | 2 |
|  | **12cii** | **Ethics** | State whether permission/licensing was obtained for the use of original, copyrighted data. | 1 |
|  | **12d** | **Protocol** | Provide a study protocol. | 8 |
|  | **12e** | **Data availability** | State where study data, code repository, and model parameters can be accessed. | 1 |
