# Supplementary material for: Evaluating the clinical utility of large language models for hepatocellular carcinoma treatment recommendations: A nationwide retrospective registry study
Source: PLoS Med. 2026 Jan 13;23(1):e1004855. doi: 10.1371/journal.pmed.1004855 (PMC12799000; doi:10.1371/journal.pmed.1004855)
Supplement: S2 Table — (DOCX) [file pmed.1004855.s016.docx]

**S2 Table. Baseline clinical characteristics according to concordance between physician decisions and Gemini 2.0-generated treatment recommendations.**

| **Clinical characteristics** | **Overall (n^1^ = 13,614)** | **Treatment concordance with Gemini** | | ***P* value^2^** |
| --- | --- | --- | --- | --- |
|  |  | **Mismatch (n^1^ = 9,168)** | **Match (n^1^ = 4,446)** |  |
| **Age at diagnosis** | 62.66 ± 11.54 | 62.39 ± 11.60 | 63.21 ± 11.40 | 0.571 |
| **Sex** |  |  |  | 0.224 |
| Male | 10,783 (79.2%) | 7,234 (78.9%) | 3,549 (79.8%) |  |
| Female | 2,831 (20.8%) | 1,934 (21.1%) | 897 (20.2%) |  |
| **Diabetes mellitus** | 3,998 (29.4%) | 2,650 (28.9%) | 1,348 (30.3%) | 0.092 |
| **Hypertension** | 5,212 (38.3%) | 3,463 (37.8%) | 1,749 (39.3%) | 0.080 |
| **Hepatitis B** | 7,526 (55.3%) | 5,065 (55.2%) | 2,461 (55.4%) | 0.912 |
| **Hepatitis C** | 1,651 (12.1%) | 1,053 (11.5%) | 598 (13.5%) | 0.001 |
| **Past smoking history** | 6,177 (45.4%) | 4,125 (45.0%) | 2,052 (46.2%) | 0.205 |
| **Past alcohol use** | 4,991 (36.7%) | 3,323 (36.2%) | 1,668 (37.5%) | 0.150 |
| **ECOG performance status** |  |  |  | < 0.001 |
| 0 | 6,773 (49.8%) | 4,566 (49.8%) | 2,207 (49.6%) |  |
| 1 | 4,032 (29.6%) | 2,767 (30.2%) | 1,265 (28.5%) |  |
| 2 | 2,442 (17.9%) | 1,712 (18.7%) | 730 (16.4%) |  |
| 3 | 231 (1.7%) | 83 (0.9%) | 148 (3.3%) |  |
| 4 | 136 (1.0%) | 40 (0.4%) | 96 (2.2%) |  |
| **Albumin (g/dL)** | 3.72 ± 0.67 | 3.75 ± 0.64 | 3.68 ± 0.72 | < 0.001 |
| **Total bilirubin (mg/dL)** | 1.62 ± 2.86 | 1.52 ± 2.53 | 1.83 ± 3.42 | 0.002 |
| **INR** | 1.16 ± 0.55 | 1.15 ± 0.64 | 1.17 ± 0.29 | 0.007 |
| **Creatinine (mg/dL)** | 0.98 ± 0.78 | 0.97 ± 0.77 | 1.00 ± 0.80 | 0.028 |
| **Sodium (mmol/L)** | 138.17 ± 5.03 | 138.28 ± 4.66 | 137.93 ± 5.70 | 0.119 |
| **ALT (IU/mL)** | 54.43 ± 100.68 | 54.43 ± 110.50 | 54.41 ± 76.53 | 0.423 |
| **Platelet (10^3^/uL)** | 164.01 ± 93.49 | 165.71 ± 94.18 | 160.51 ± 91.95 | 0.125 |
| **AFP (ng/mL)** | 13,409.30 ± 104,384.94 | 13,984.64 ± 105,592.45 | 12,222.90 ± 101,851.25 | 0.913 |
| **Multiple tumors** | 5,532 (40.6%) | 3,430 (37.4%) | 2,102 (47.3%) | < 0.001 |
| **Maximum tumor diameter (cm)** | 4.70 ± 3.91 | 4.78 ± 3.93 | 4.56 ± 3.86 | 0.691 |
| **Portal vein invasion** | 3,298 (24.2%) | 2,472 (27.0%) | 826 (18.6%) | < 0.001 |
| **Hepatic vein invasion** | 773 (5.7%) | 560 (6.1%) | 213 (4.8%) | 0.002 |
| **Bile duct invasion** | 366 (2.7%) | 275 (3.0%) | 91 (2.0%) | 0.001 |
| **Hepatic artery invasion** | 158 (1.2%) | 115 (1.3%) | 43 (1.0%) | 0.148 |
| **Lymph node metastasis** | 976 (7.2%) | 684 (7.5%) | 292 (6.6%) | 0.060 |
| **Extrahepatic metastasis** | 1,457 (10.7%) | 979 (10.7%) | 478 (10.8%) | 0.906 |
| **Ascites** |  |  |  | < 0.001 |
| None | 10,098 (74.2%) | 6,850 (74.7%) | 3,248 (73.1%) |  |
| Mild | 2,217 (16.3%) | 1,540 (16.8%) | 677 (15.2%) |  |
| Moderate to severe | 1,299 (9.5%) | 778 (8.5%) | 521 (11.7%) |  |
| **Hepatic encephalopathy grade** |  |  |  | 0.273 |
| None | 13,277 (97.5%) | 8,931 (97.4%) | 4,346 (97.8%) |  |
| Grade 1 or 2 | 266 (2.0%) | 191 (2.1%) | 75 (1.7%) |  |
| Grade 3 or 4 | 71 (0.5%) | 46 (0.5%) | 25 (0.6%) |  |
| **Child-Pugh classification** |  |  |  | < 0.001 |
| A | 11,240 (82.6%) | 7,663 (83.6%) | 3,577 (80.5%) |  |
| B | 2,241 (16.5%) | 1,463 (16.0%) | 778 (17.5%) |  |
| C | 133 (1.0%) | 42 (0.5%) | 91 (2.0%) |  |
| **BCLC stage** |  |  |  | < 0.001 |
| A | 4,064 (29.9%) | 2,729 (29.8%) | 1,335 (30.0%) |  |
| B | 5,265 (38.7%) | 3,244 (35.4%) | 2,021 (45.5%) |  |
| C | 4,285 (31.5%) | 3,195 (34.8%) | 1,090 (24.5%) |  |
| **MELD score** | 9.81 ± 4.05 | 9.68 ± 3.72 | 10.10 ± 4.63 | < 0.001 |

^1^n (%); Mean ± SD, ^2^Fisher’s exact test

ECOG, Eastern Cooperative Oncology Group; INR, international normalized ratio; ALT, Alanine aminotransferase; AFP, alpha-fetoprotein; BCLC, Barcelona clinic liver cancer; MELD, model for end-stage liver disease.
