# Supplementary material for: Evaluating the clinical utility of large language models for hepatocellular carcinoma treatment recommendations: A nationwide retrospective registry study
Source: PLoS Med. 2026 Jan 13;23(1):e1004855. doi: 10.1371/journal.pmed.1004855 (PMC12799000; doi:10.1371/journal.pmed.1004855)
Supplement: S3 Table — (DOCX) [file pmed.1004855.s017.docx]

**S3 Table. Baseline clinical characteristics according to concordance between physician decisions and Claude 3.5-generated treatment recommendations.**

| **Clinical characteristics** | **Overall (n^1^ = 13,614)** | **Treatment concordance with Claude** | | ***P* value^2^** |
| --- | --- | --- | --- | --- |
|  |  | **Mismatch (n^1^ = 9,973)** | **Match (n^1^ = 3,641)** |  |
| **Age at diagnosis** | 62.66 ± 11.54 | 62.62 ± 11.60 | 62.76 ± 11.39 | 0.977 |
| **Sex** |  |  |  | 0.182 |
| Male | 10,783 (79.2%) | 7,871 (78.9%) | 2,912 (80.0%) |  |
| Female | 2,831 (20.8%) | 2,102 (21.1%) | 729 (20.0%) |  |
| **Diabetes mellitus** | 3,998 (29.4%) | 2,905 (29.1%) | 1,093 (30.0%) | 0.318 |
| **Hypertension** | 5,212 (38.3%) | 3,829 (38.4%) | 1,383 (38.0%) | 0.676 |
| **Hepatitis B** | 7,526 (55.3%) | 5,512 (55.3%) | 2,014 (55.3%) | 0.969 |
| **Hepatitis C** | 1,651 (12.1%) | 1,172 (11.8%) | 479 (13.2%) | 0.028 |
| **Past smoking history** | 6,177 (45.4%) | 4,502 (45.1%) | 1,675 (46.0%) | 0.371 |
| **Past alcohol use** | 4,991 (36.7%) | 3,604 (36.1%) | 1,387 (38.1%) | 0.037 |
| **ECOG performance status** |  |  |  | < 0.001 |
| 0 | 6,773 (49.8%) | 4,965 (49.8%) | 1,808 (49.7%) |  |
| 1 | 4,032 (29.6%) | 3,023 (30.3%) | 1,009 (27.7%) |  |
| 2 | 2,442 (17.9%) | 1,858 (18.6%) | 584 (16.0%) |  |
| 3 | 231 (1.7%) | 88 (0.9%) | 143 (3.9%) |  |
| 4 | 136 (1.0%) | 39 (0.4%) | 97 (2.7%) |  |
| **Albumin (g/dL)** | 3.72 ± 0.67 | 3.74 ± 0.66 | 3.69 ± 0.69 | 0.820 |
| **Total bilirubin (mg/dL)** | 1.62 ± 2.86 | 1.41 ± 1.96 | 2.21 ± 4.42 | < 0.001 |
| **INR** | 1.16 ± 0.55 | 1.15 ± 0.61 | 1.18 ± 0.31 | < 0.001 |
| **Creatinine (mg/dL)** | 0.98 ± 0.78 | 0.97 ± 0.79 | 1.00 ± 0.75 | < 0.001 |
| **Sodium (mmol/L)** | 138.17 ± 5.03 | 138.23 ± 4.96 | 137.98 ± 5.20 | 0.158 |
| **ALT (IU/mL)** | 54.43 ± 100.68 | 53.47 ± 105.18 | 57.06 ± 87.12 | 0.233 |
| **Platelet (10^3^/uL)** | 164.01 ± 93.49 | 166.11 ± 93.69 | 158.26 ± 92.70 | 0.454 |
| **AFP (ng/mL)** | 13,409.30 ± 104,384.94 | 13,774.47 ± 107,230.59 | 12,409.06 ± 96,167.17 | 0.900 |
| **Multiple tumors** | 5,532 (40.6%) | 3,784 (37.9%) | 1,748 (48.0%) | < 0.001 |
| **Maximum tumor diameter (cm)** | 4.70 ± 3.91 | 4.79 ± 3.95 | 4.46 ± 3.79 | 0.605 |
| **Portal vein invasion** | 3,298 (24.2%) | 2,650 (26.6%) | 648 (17.8%) | < 0.001 |
| **Hepatic vein invasion** | 773 (5.7%) | 596 (6.0%) | 177 (4.9%) | 0.014 |
| **Bile duct invasion** | 366 (2.7%) | 283 (2.8%) | 83 (2.3%) | 0.082 |
| **Hepatic artery invasion** | 158 (1.2%) | 131 (1.3%) | 27 (0.7%) | 0.005 |
| **Lymph node metastasis** | 976 (7.2%) | 763 (7.7%) | 213 (5.9%) | < 0.001 |
| **Extrahepatic metastasis** | 1,457 (10.7%) | 1,091 (10.9%) | 366 (10.1%) | 0.141 |
| **Ascites** |  |  |  | 0.006 |
| None | 10,098 (74.2%) | 7,426 (74.5%) | 2,672 (73.4%) |  |
| Mild | 2,217 (16.3%) | 1,645 (16.5%) | 572 (15.7%) |  |
| Moderate to severe | 1,299 (9.5%) | 902 (9.0%) | 397 (10.9%) |  |
| **Hepatic encephalopathy grade** |  |  |  | 0.708 |
| None | 13,277 (97.5%) | 9,729 (97.6%) | 3,548 (97.4%) |  |
| Grade 1 or 2 | 266 (2.0%) | 195 (2.0%) | 71 (2.0%) |  |
| Grade 3 or 4 | 71 (0.5%) | 49 (0.5%) | 22 (0.6%) |  |
| **Child-Pugh classification** |  |  |  | < 0.001 |
| A | 11,240 (82.6%) | 8,304 (83.3%) | 2,936 (80.6%) |  |
| B | 2,241 (16.5%) | 1,626 (16.3%) | 615 (16.9%) |  |
| C | 133 (1.0%) | 43 (0.4%) | 90 (2.5%) |  |
| **BCLC stage** |  |  |  | < 0.001 |
| A | 4,064 (29.9%) | 3,006 (30.1%) | 1,058 (29.1%) |  |
| B | 5,265 (38.7%) | 3,524 (35.3%) | 1,741 (47.8%) |  |
| C | 4,285 (31.5%) | 3,443 (34.5%) | 842 (23.1%) |  |
| **MELD score** | 9.81 ± 4.05 | 9.59 ± 3.53 | 10.43 ± 5.15 | < 0.001 |

^1^n (%); Mean ± SD, ^2^Fisher’s exact test

ECOG, Eastern Cooperative Oncology Group; INR, international normalized ratio; ALT, Alanine aminotransferase; AFP, alpha-fetoprotein; BCLC, Barcelona clinic liver cancer; MELD, model for end-stage liver disease.
