# Supplementary material for: Evaluating the clinical utility of large language models for hepatocellular carcinoma treatment recommendations: A nationwide retrospective registry study
Source: PLoS Med. 2026 Jan 13;23(1):e1004855. doi: 10.1371/journal.pmed.1004855 (PMC12799000; doi:10.1371/journal.pmed.1004855)
Supplement: S4 Table — (DOCX) [file pmed.1004855.s018.docx]

**S4 Table. Univariate analyses of overall survival in HCC patients according to BCLC stage.**

| **Clinical characteristics** | **BCLC stage A** | | | **BCLC stage B** | | | **BCLC stage C** | | |
| --- | --- | --- | --- | --- | --- | --- | --- | --- | --- |
|  | **HR** | **95% CI** | ***P* value** | **HR** | **95% CI** | ***P* value** | **HR** | **95% CI** | ***P* value** |
| **Age at diagnosis** | 1.018 | 1.014, 1.023 | < 0.001 | 1.016 | 1.013, 1.019 | < 0.001 | 1.001 | 0.998, 1.003 | 0.703 |
| **Male** | 1.028 | 0.936, 1.129 | 0.567 | 1.095 | 1.012, 1.185 | 0.024 | 0.950 | 0.872, 1.034 | 0.232 |
| **Diabetes mellitus** | 1.087 | 0.995, 1.187 | 0.063 | 1.078 | 1.007, 1.153 | 0.031 | 1.007 | 0.938, 1.081 | 0.848 |
| **Hypertension** | 1.035 | 0.950, 1.128 | 0.431 | 1.025 | 0.961, 1.093 | 0.454 | 0.957 | 0.896, 1.022 | 0.190 |
| **Hepatitis B** | 0.675 | 0.622, 0.734 | < 0.001 | 0.831 | 0.780, 0.886 | < 0.001 | 1.065 | 1.000, 1.134 | 0.049 |
| **Hepatitis C** | 1.283 | 1.148, 1.434 | < 0.001 | 1.123 | 1.026, 1.229 | 0.012 | 0.964 | 0.866, 1.072 | 0.494 |
| **Past smoking history** | 1.070 | 0.986, 1.164 | 0.116 | 0.926 | 0.868, 0.987 | 0.019 | 1.059 | 0.995, 1.126 | 0.070 |
| **Past alcohol use** | 1.098 | 1.005, 1.199 | 0.039 | 1.001 | 0.937, 1.070 | 0.978 | 0.998 | 0.937, 1.062 | 0.939 |
| **ECOG performance status** | 1.332 | 1.265, 1.403 | < 0.001 | 1.277 | 1.230, 1.326 | < 0.001 | 1.310 | 1.268, 1.354 | < 0.001 |
| **Albumin (g/dL)** | 0.521 | 0.492, 0.553 | < 0.001 | 0.562 | 0.535, 0.591 | < 0.001 | 0.637 | 0.610, 0.665 | < 0.001 |
| **Total bilirubin (mg/dL)** | 1.089 | 1.073, 1.104 | < 0.001 | 1.101 | 1.085, 1.117 | < 0.001 | 1.045 | 1.039, 1.051 | < 0.001 |
| **INR** | 2.989 | 2.628, 3.399 | < 0.001 | 2.099 | 1.896, 2.325 | < 0.001 | 1.071 | 1.052, 1.090 | < 0.001 |
| **Creatinine (mg/dL)** | 1.085 | 1.047, 1.125 | < 0.001 | 1.084 | 1.047, 1.121 | < 0.001 | 1.118 | 1.074, 1.164 | < 0.001 |
| **Sodium (mmol/L)** | 0.984 | 0.981, 0.987 | < 0.001 | 0.983 | 0.979, 0.986 | < 0.001 | 0.931 | 0.925, 0.937 | < 0.001 |
| **ALT (IU/mL)** | 1.000 | 0.999, 1.001 | 0.469 | 1.001 | 1.001, 1.001 | < 0.001 | 1.000 | 1.000, 1.001 | 0.009 |
| **Platelet (10^3^/uL)** | 0.997 | 0.997, 0.998 | < 0.001 | 1.001 | 1.000, 1.001 | < 0.001 | 1.001 | 1.001, 1.001 | < 0.001 |
| **AFP (ng/mL)** | 1.000 | 1.000, 1.000 | 0.841 | 1.000 | 1.000, 1.000 | < 0.001 | 1.000 | 1.000, 1.000 | < 0.001 |
| **Multiple tumors** | - | - | - | 1.245 | 1.167, 1.328 | < 0.001 | 1.336 | 1.254, 1.423 | < 0.001 |
| **Maximum tumor diameter (cm)** | 1.156 | 1.083, 1.234 | < 0.001 | 1.086 | 1.076, 1.096 | < 0.001 | 1.023 | 1.017, 1.031 | < 0.001 |
| **Portal vein invasion** | - | - | - | - | - | - | 1.300 | 1.213, 1.394 | < 0.001 |
| **Hepatic vein invasion** | - | - | - | - | - | - | 1.156 | 1.066, 1.254 | < 0.001 |
| **Bile duct invasion** | - | - | - | - | - | - | 0.818 | 0.729, 0.918 | < 0.001 |
| **Hepatic artery invasion** | - | - | - | - | - | - | 0.829 | 0.697, 0.987 | 0.035 |
| **Lymph node metastasis** | - | - | - | - | - | - | 1.469 | 1.363, 1.582 | < 0.001 |
| **Extrahepatic metastasis** | - | - | - | - | - | - | 1.735 | 1.624, 1.853 | < 0.001 |
| **Ascites** | 1.934 | 1.803, 2.074 | < 0.001 | 1.932 | 1.819, 2.052 | < 0.001 | 1.576 | 1.517, 1.637 | < 0.001 |
| **Hepatic encephalopathy grade** | 2.123 | 1.807, 2.493 | < 0.001 | 1.269 | 1.052, 1.529 | 0.013 | 1.169 | 1.034, 1.321 | 0.012 |
| **Child-Pugh classification** | 7.674 | 4.982, 11.818 | < 0.001 | 34.566 | 4.814, 248.205 | < 0.001 | 1.565 | 1.274, 1.923 | < 0.001 |
| **MELD score** | 1.103 | 1.093, 1.114 | < 0.001 | 1.091 | 1.081, 1.102 | < 0.001 | 1.059 | 1.054, 1.065 | < 0.001 |
| **ChatGPT 4o-matched decision** | 0.626 | 0.562, 0.697 | < 0.001 | 1.091 | 1.023, 1.164 | 0.008 | 2.271 | 2.110, 2.443 | < 0.001 |
| **Gemini 2.0-matched decision** | 0.733 | 0.668, 0.804 | < 0.001 | 1.162 | 1.090, 1.240 | < 0.001 | 2.261 | 2.104, 2.430 | < 0.001 |
| **Claude 3.5-matched decision** | 0.751 | 0.679, 0.830 | < 0.001 | 1.135 | 1.062, 1.213 | < 0.001 | 2.153 | 1.991, 2.327 | < 0.001 |

HCC, hepatocellular carcinoma; BCLC, Barcelona clinic liver cancer; HR, hazard ratio; CI, confidence interval; ECOG, Eastern Cooperative Oncology Group; INR, international normalized ratio; ALT, Alanine aminotransferase; AFP, alpha-fetoprotein; MELD, model for end-stage liver disease. *P* values were calculated from univariable Cox proportional hazards models.
