# Supplementary material for: Evaluating the clinical utility of large language models for hepatocellular carcinoma treatment recommendations: A nationwide retrospective registry study
Source: PLoS Med. 2026 Jan 13;23(1):e1004855. doi: 10.1371/journal.pmed.1004855 (PMC12799000; doi:10.1371/journal.pmed.1004855)
Supplement: S5 Table — (DOCX) [file pmed.1004855.s019.docx]

**S5 Table. Era-stratified sensitivity analysis of survival outcomes by BCLC stage and LLM model.**

| **LLM** | **BCLC stage** | **Era** | **HR** | **95% CI** | ***P* value** |
| --- | --- | --- | --- | --- | --- |
| **ChatGPT 4o** | **A** | 2008-2012 | 0.828 | 0.710-0.967 | 0.017 |
|  |  | 2013-2016 | 0.628 | 0.519-0.759 | < 0.001 |
|  |  | 2017-2020 | 0.422 | 0.329-0.541 | < 0.001 |
|  | **B** | 2008-2012 | 1.042 | 0.946-1.147 | 0.405 |
|  |  | 2013-2016 | 1.059 | 0.948-1.183 | 0.310 |
|  |  | 2017-2020 | 1.077 | 0.934-1.241 | 0.307 |
|  | **C** | 2008-2012 | 2.220 | 1.967-2.506 | < 0.001 |
|  |  | 2013-2016 | 1.998 | 1.769-2.256 | < 0.001 |
|  |  | 2017-2020 | 2.831 | 2.458-3.260 | < 0.001 |
| **Gemini 2.0** | **A** | 2008-2012 | 0.753 | 0.659-0.860 | < 0.001 |
|  |  | 2013-2016 | 0.768 | 0.654-0.902 | 0.001 |
|  |  | 2017-2020 | 0.730 | 0.587-0.908 | 0.005 |
|  | **B** | 2008-2012 | 1.061 | 0.964-1.169 | 0.228 |
|  |  | 2013-2016 | 1.159 | 1.037-1.295 | 0.010 |
|  |  | 2017-2020 | 1.240 | 1.077-1.427 | 0.003 |
|  | **C** | 2008-2012 | 2.484 | 2.189-2.820 | < 0.001 |
|  |  | 2013-2016 | 2.151 | 1.905-2.429 | < 0.001 |
|  |  | 2017-2020 | 2.411 | 2.117-2.745 | < 0.001 |
| **Claude 3.5** | **A** | 2008-2012 | 0.809 | 0.700-0.934 | 0.004 |
|  |  | 2013-2016 | 0.726 | 0.609-0.864 | < 0.001 |
|  |  | 2017-2020 | 0.716 | 0.565-0.909 | 0.006 |
|  | **B** | 2008-2012 | 1.073 | 0.972-1.185 | 0.160 |
|  |  | 2013-2016 | 1.118 | 0.997-1.252 | 0.056 |
|  |  | 2017-2020 | 1.159 | 1.001-1.343 | 0.049 |
|  | **C** | 2008-2012 | 2.076 | 1.812-2.379 | < 0.001 |
|  |  | 2013-2016 | 2.012 | 1.774-2.281 | < 0.001 |
|  |  | 2017-2020 | 2.641 | 2.271-3.071 | < 0.001 |

LLM, large language model; BCLC, Barcelona clinic liver cancer; HR, hazard ratio; CI, confidence interval. *P* values were calculated from univariable Cox proportional hazards models.
