# Supplementary material for: Evaluating the clinical utility of large language models for hepatocellular carcinoma treatment recommendations: A nationwide retrospective registry study
Source: PLoS Med. 2026 Jan 13;23(1):e1004855. doi: 10.1371/journal.pmed.1004855 (PMC12799000; doi:10.1371/journal.pmed.1004855)
Supplement: S6 Table — (DOCX) [file pmed.1004855.s020.docx]

**S6 Table. Inverse probability weighted cox proportional hazards model for overall survival according to LLM recommendation adherence (BCLC Stage A and C).**

| **Clinical characteristics** | **BCLC stage A** | | | **BCLC stage C** | | |
| --- | --- | --- | --- | --- | --- | --- |
|  | **HR** | **95% CI** | ***P* value** | **HR** | **95% CI** | ***P* value** |
| **ChatGPT 4o-matched decision** | 0.847 | 0.754, 0.951 | 0.005 | 1.513 | 1.369, 1.672 | < 0.001 |
| **Gemini 2.0-matched decision** | 0.869 | 0.791, 0.955 | 0.004 | 1.518 | 1.396, 1.652 | < 0.001 |
| **Claude 3.5-matched decision** | 0.846 | 0.767, 0.934 | 0.001 | 1.464 | 1.339, 1.601 | < 0.001 |

BCLC, Barcelona clinic liver cancer; HR, hazard ratio; CI, confidence interval. *P* values were calculated from IPTW-adjusted Cox proportional hazards models.
