# Supplementary material for: Evaluating the clinical utility of large language models for hepatocellular carcinoma treatment recommendations: A nationwide retrospective registry study
Source: PLoS Med. 2026 Jan 13;23(1):e1004855. doi: 10.1371/journal.pmed.1004855 (PMC12799000; doi:10.1371/journal.pmed.1004855)
Supplement: S7 Table — (DOCX) [file pmed.1004855.s021.docx]

**S7 Table. Doubly-robust (Augmented IPTW) cox proportional hazards model for overall survival according to LLM recommendation adherence.**

| **Clinical characteristics** | **BCLC stage A** | | | **BCLC stage C** | | |
| --- | --- | --- | --- | --- | --- | --- |
|  | **HR** | **95% CI** | ***P* value** | **HR** | **95% CI** | ***P* value** |
| **ChatGPT 4o-matched decision** | 0.789 | 0.693, 0.899 | < 0.001 | 1.571 | 1.429, 1.727 | < 0.001 |
| **Gemini 2.0-matched decision** | 0.957 | 0.862, 1.062 | 0.408 | 1.495 | 1.380, 1.620 | < 0.001 |
| **Claude 3.5-matched decision** | 0.861 | 0.769, 0.963 | 0.001 | 1.383 | 1.265, 1.512 | < 0.001 |

BCLC, Barcelona clinic liver cancer; HR, hazard ratio; CI, confidence interval. *P* values were calculated from doubly-robust (augmented IPTW) Cox proportional hazards models.
