# Supplementary material for: Evaluating the clinical utility of large language models for hepatocellular carcinoma treatment recommendations: A nationwide retrospective registry study
Source: PLoS Med. 2026 Jan 13;23(1):e1004855. doi: 10.1371/journal.pmed.1004855 (PMC12799000; doi:10.1371/journal.pmed.1004855)
Supplement: S8 Table — (DOCX) [file pmed.1004855.s022.docx]

**S8 Table. Baseline clinical characteristics according to concordance between physician decisions and ChatGPT 4o-generated treatment recommendations in BCLC stage A.**

| **Clinical characteristics** | **Overall (n^1^ = 4,064)** | **Treatment concordance with ChatGPT** | | ***P* value^2^** |
| --- | --- | --- | --- | --- |
|  |  | **Mismatch (n^1^ = 2,923)** | **Match (n^1^ = 1,141)** |  |
| **Age at diagnosis** | 62.56 ± 10.56 | 63.52 ± 10.61 | 60.09 ± 10.04 | < 0.001 |
| **Sex** |  |  |  | 0.004 |
| Male | 2,967 (73.0%) | 2,097 (71.7%) | 870 (76.2%) |  |
| Female | 1,097 (27.0%) | 826 (28.3%) | 271 (23.8%) |  |
| **Diabetes mellitus** | 1,232 (30.3%) | 909 (31.1%) | 323 (28.3%) | 0.087 |
| **Hypertension** | 1,497 (36.8%) | 1,085 (37.1%) | 412 (36.1%) | 0.563 |
| **Hepatitis B** | 2,265 (55.7%) | 1,507 (51.6%) | 758 (66.4%) | < 0.001 |
| **Hepatitis C** | 546 (13.4%) | 439 (15.0%) | 107 (9.4%) | < 0.001 |
| **Past smoking history** | 1,635 (40.2%) | 1,136 (38.9%) | 499 (43.7%) | 0.005 |
| **Past alcohol use** | 1,307 (32.2%) | 952 (32.6%) | 355 (31.1%) | 0.390 |
| **Albumin (g/dL)** | 3.86 ± 0.65 | 3.78 ± 0.65 | 4.08 ± 0.60 | < 0.001 |
| **Total bilirubin (mg/dL)** | 1.30 ± 1.87 | 1.39 ± 2.05 | 1.05 ± 1.29 | 0.480 |
| **INR** | 1.15 ± 0.21 | 1.17 ± 0.20 | 1.10 ± 0.20 | < 0.001 |
| **Creatinine (mg/dL)** | 0.97 ± 0.87 | 0.97 ± 0.84 | 0.97 ± 0.94 | 0.038 |
| **Sodium (mmol/L)** | 139.08 ± 5.44 | 138.91 ± 5.64 | 139.54 ± 4.89 | 0.080 |
| **ALT (IU/mL)** | 40.12 ± 52.34 | 40.07 ± 54.02 | 40.23 ± 47.78 | 0.152 |
| **Platelet (10^3^/uL)** | 132.57 ± 64.82 | 124.46 ± 65.06 | 153.35 ± 59.35 | < 0.001 |
| **AFP (ng/mL)** | 1,741.52 ± 83,226.43 | 2,186.84 ± 98,002.33 | 600.70 ± 8,199.56 | 0.681 |
| **Maximum tumor diameter (cm)** | 1.91 ± 0.63 | 1.85 ± 0.63 | 2.08 ± 0.60 | < 0.001 |
| **MELD score** | 9.38 ± 3.55 | 9.70 ± 3.65 | 8.57 ± 3.14 | < 0.001 |

^1^n (%); Mean ± SD, ^2^Fisher’s exact test

INR, international normalized ratio; ALT, Alanine aminotransferase; AFP, alpha-fetoprotein; MELD, model for end-stage liver disease.
