# Supplementary material for: Evaluating the clinical utility of large language models for hepatocellular carcinoma treatment recommendations: A nationwide retrospective registry study
Source: PLoS Med. 2026 Jan 13;23(1):e1004855. doi: 10.1371/journal.pmed.1004855 (PMC12799000; doi:10.1371/journal.pmed.1004855)
Supplement: S9 Table — (DOCX) [file pmed.1004855.s023.docx]

**S9 Table. Baseline clinical characteristics according to concordance between physician decisions and ChatGPT 4o-generated treatment recommendations in BCLC stage C.**

| **Clinical characteristics** | **Overall (n^1^ = 4,285)** | **Treatment concordance with ChatGPT** | | ***P* value^2^** |
| --- | --- | --- | --- | --- |
|  |  | **Mismatch (n^1^ = 3,270)** | **Match (n^1^ = 1,015)** |  |
| **Age at diagnosis** | 60.59 ± 12.17 | 59.88 ± 11.92 | 62.88 ± 12.67 | < 0.001 |
| **Sex** |  |  |  | 0.048 |
| Male | 3,614 (84.3%) | 2,778 (85.0%) | 836 (82.4%) |  |
| Female | 671 (15.7%) | 492 (15.0%) | 179 (17.6%) |  |
| **Diabetes mellitus** | 1,057 (24.7%) | 810 (24.8%) | 247 (24.3%) | 0.803 |
| **Hypertension** | 1,389 (32.4%) | 1,053 (32.2%) | 336 (33.1%) | 0.591 |
| **Hepatitis B** | 2,602 (60.7%) | 2,036 (62.3%) | 566 (55.8%) | < 0.001 |
| **Hepatitis C** | 396 (9.2%) | 292 (8.9%) | 104 (10.2%) | 0.215 |
| **Past smoking history** | 2,228 (52.0%) | 1,723 (52.7%) | 505 (49.8%) | 0.106 |
| **Past alcohol use** | 1,779 (41.5%) | 1,369 (41.9%) | 410 (40.4%) | 0.423 |
| **ECOG performance status** |  |  |  | < 0.001 |
| 0 | 1,540 (35.9%) | 1,258 (38.5%) | 282 (27.8%) |  |
| 1 | 1,566 (36.5%) | 1,201 (36.7%) | 365 (36.0%) |  |
| 2 | 940 (21.9%) | 678 (20.7%) | 262 (25.8%) |  |
| 3 | 149 (3.5%) | 84 (2.6%) | 65 (6.4%) |  |
| 4 | 90 (2.1%) | 49 (1.5%) | 41 (4.0%) |  |
| **Albumin (g/dL)** | 3.48 ± 0.67 | 3.54 ± 0.66 | 3.27 ± 0.66 | < 0.001 |
| **Total bilirubin (mg/dL)** | 2.56 ± 4.30 | 2.28 ± 3.92 | 3.47 ± 5.23 | < 0.001 |
| **INR** | 1.22 ± 0.93 | 1.21 ± 1.05 | 1.27 ± 0.33 | < 0.001 |
| **Creatinine (mg/dL)** | 0.97 ± 0.66 | 0.95 ± 0.65 | 1.02 ± 0.70 | 0.004 |
| **Sodium (mmol/L)** | 136.61 ± 4.63 | 137.03 ± 4.51 | 135.25 ± 4.75 | < 0.001 |
| **ALT (IU/mL)** | 74.60 ± 151.77 | 72.05 ± 160.41 | 82.82 ± 119.45 | 0.050 |
| **Platelet (10^3^/uL)** | 191.87 ± 110.20 | 186.37 ± 101.75 | 209.60 ± 132.36 | 0.003 |
| **AFP (ng/mL)** | 35,041.52 ± 157,429.50 | 33,650.25 ± 150,022.22 | 39,523.75 ± 179,231.97 | 0.826 |
| **Multiple tumors** | 2,617 (61.1%) | 1,920 (58.7%) | 697 (68.7%) | < 0.001 |
| **Maximum tumor diameter (cm)** | 7.00 ± 4.64 | 6.86 ± 4.49 | 7.48 ± 5.06 | < 0.001 |
| **Portal vein invasion** | 3,119 (72.8%) | 2,538 (77.6%) | 581 (57.2%) | < 0.001 |
| **Hepatic vein invasion** | 747 (17.4%) | 543 (16.6%) | 204 (20.1%) | 0.012 |
| **Bile duct invasion** | 339 (7.9%) | 249 (7.6%) | 90 (8.9%) | 0.206 |
| **Hepatic artery invasion** | 141 (3.3%) | 101 (3.1%) | 40 (3.9%) | 0.191 |
| **Lymph node metastasis** | 921 (21.5%) | 590 (18.0%) | 331 (32.6%) | < 0.001 |
| **Extrahepatic metastasis** | 1,408 (32.9%) | 775 (23.7%) | 633 (62.4%) | < 0.001 |
| **Ascites** |  |  |  | < 0.001 |
| None | 2,192 (51.2%) | 1,808 (55.3%) | 384 (37.8%) |  |
| Mild | 1,188 (27.7%) | 876 (26.8%) | 312 (30.7%) |  |
| Moderate to severe | 905 (21.1%) | 586 (17.9%) | 319 (31.4%) |  |
| **Hepatic encephalopathy grade** |  |  |  | 0.042 |
| None | 4,127 (96.3%) | 3,162 (96.7%) | 965 (95.1%) |  |
| Grade 1 or 2 | 120 (2.8%) | 84 (2.6%) | 36 (3.5%) |  |
| Grade 3 or 4 | 38 (0.9%) | 24 (0.7%) | 14 (1.4%) |  |
| **Child-Pugh classification** |  |  |  | < 0.001 |
| A | 2,981 (69.6%) | 2,422 (74.1%) | 559 (55.1%) |  |
| B | 1,200 (28.0%) | 790 (24.2%) | 410 (40.4%) |  |
| C | 104 (2.4%) | 58 (1.8%) | 46 (4.5%) |  |
| **MELD score** | 11.21 ± 5.02 | 10.73 ± 4.64 | 12.78 ± 5.81 | < 0.001 |

^1^n (%); Mean ± SD, ^2^Fisher’s exact test

ECOG, Eastern Cooperative Oncology Group; INR, international normalized ratio; ALT, Alanine aminotransferase; AFP, alpha-fetoprotein; BCLC, Barcelona clinic liver cancer; MELD, model for end-stage liver disease.
