# Supplementary material for: Evaluating the clinical utility of large language models for hepatocellular carcinoma treatment recommendations: A nationwide retrospective registry study
Source: PLoS Med. 2026 Jan 13;23(1):e1004855. doi: 10.1371/journal.pmed.1004855 (PMC12799000; doi:10.1371/journal.pmed.1004855)
Supplement: S10 Table — (DOCX) [file pmed.1004855.s024.docx]

**S10 Table. Baseline clinical characteristics according to concordance between physician decisions and Gemini 2.0-generated treatment recommendations in BCLC stage A.**

| **Clinical characteristics** | **Overall (n^1^ = 4,064)** | **Treatment concordance with Gemini** | | ***P* value^2^** |
| --- | --- | --- | --- | --- |
|  |  | **Mismatch (n^1^ = 2,729)** | **Match (n^1^ = 1,335)** |  |
| **Age at diagnosis** | 62.56 ± 10.56 | 62.85 ± 10.67 | 61.97 ± 10.31 | 0.404 |
| **Sex** |  |  |  | 0.071 |
| Male | 2,967 (73.0%) | 1,968 (72.1%) | 999 (74.8%) |  |
| Female | 1,097 (27.0%) | 761 (27.9%) | 336 (25.2%) |  |
| **Diabetes mellitus** | 1,232 (30.3%) | 841 (30.8%) | 391 (29.3%) | 0.327 |
| **Hypertension** | 1,497 (36.8%) | 987 (36.2%) | 510 (38.2%) | 0.213 |
| **Hepatitis B** | 2,265 (55.7%) | 1,465 (53.7%) | 800 (59.9%) | < 0.001 |
| **Hepatitis C** | 546 (13.4%) | 384 (14.1%) | 162 (12.1%) | 0.096 |
| **Past smoking history** | 1,635 (40.2%) | 1,104 (40.5%) | 531 (39.8%) | 0.683 |
| **Past alcohol use** | 1,307 (32.2%) | 882 (32.3%) | 425 (31.8%) | 0.775 |
| **Albumin (g/dL)** | 3.86 ± 0.65 | 3.80 ± 0.65 | 4.00 ± 0.64 | < 0.001 |
| **Total bilirubin (mg/dL)** | 1.30 ± 1.87 | 1.30 ± 1.58 | 1.29 ± 2.36 | 0.071 |
| **INR** | 1.15 ± 0.21 | 1.16 ± 0.19 | 1.13 ± 0.23 | < 0.001 |
| **Creatinine (mg/dL)** | 0.97 ± 0.87 | 0.97 ± 0.86 | 0.97 ± 0.89 | 0.062 |
| **Sodium (mmol/L)** | 139.08 ± 5.44 | 138.96 ± 5.21 | 139.33 ± 5.89 | 0.288 |
| **ALT (IU/mL)** | 40.12 ± 52.34 | 40.24 ± 54.61 | 39.86 ± 47.38 | 0.507 |
| **Platelet (10^3^/uL)** | 132.57 ± 64.82 | 127.09 ± 65.28 | 143.78 ± 62.39 | < 0.001 |
| **AFP (ng/mL)** | 1,741.52 ± 83,226.43 | 2,378.77 ± 101,455.15 | 438.85 ± 6,698.28 | 0.942 |
| **Maximum tumor diameter (cm)** | 1.91 ± 0.63 | 1.89 ± 0.62 | 1.96 ± 0.65 | < 0.001 |
| **MELD score** | 9.38 ± 3.55 | 9.57 ± 3.46 | 9.00 ± 3.71 | < 0.001 |

^1^n (%); Mean ± SD, ^2^Fisher’s exact test

INR, international normalized ratio; ALT, Alanine aminotransferase; AFP, alpha-fetoprotein; MELD, model for end-stage liver disease.
