# Supplementary material for: Evaluating the clinical utility of large language models for hepatocellular carcinoma treatment recommendations: A nationwide retrospective registry study
Source: PLoS Med. 2026 Jan 13;23(1):e1004855. doi: 10.1371/journal.pmed.1004855 (PMC12799000; doi:10.1371/journal.pmed.1004855)
Supplement: S11 Table — (DOCX) [file pmed.1004855.s025.docx]

**S11 Table. Baseline clinical characteristics according to concordance between physician decisions and Gemini 2.0-generated treatment recommendations in BCLC stage C.**

| **Clinical characteristics** | **Overall (n^1^ = 4,285)** | **Treatment concordance with Gemini** | | ***P* value^2^** |
| --- | --- | --- | --- | --- |
|  |  | **Mismatch (n^1^ = 3,195)** | **Match (n^1^ = 1,090)** |  |
| **Age at diagnosis** | 60.59 ± 12.17 | 60.07 ± 12.01 | 62.11 ± 12.50 | 0.023 |
| **Sex** |  |  |  | 0.810 |
| Male | 3,614 (84.3%) | 2,692 (84.3%) | 922 (84.6%) |  |
| Female | 671 (15.7%) | 503 (15.7%) | 168 (15.4%) |  |
| **Diabetes mellitus** | 1,057 (24.7%) | 767 (24.0%) | 290 (26.6%) | 0.088 |
| **Hypertension** | 1,389 (32.4%) | 1,039 (32.5%) | 350 (32.1%) | 0.822 |
| **Hepatitis B** | 2,602 (60.7%) | 1,970 (61.7%) | 632 (58.0%) | 0.034 |
| **Hepatitis C** | 396 (9.2%) | 267 (8.4%) | 129 (11.8%) | 0.001 |
| **Past smoking history** | 2,228 (52.0%) | 1,653 (51.7%) | 575 (52.8%) | 0.574 |
| **Past alcohol use** | 1,779 (41.5%) | 1,308 (40.9%) | 471 (43.2%) | 0.200 |
| **ECOG performance status** |  |  |  | < 0.001 |
| 0 | 1,540 (35.9%) | 1,264 (39.6%) | 276 (25.3%) |  |
| 1 | 1,566 (36.5%) | 1,170 (36.6%) | 396 (36.3%) |  |
| 2 | 940 (21.9%) | 705 (22.1%) | 235 (21.6%) |  |
| 3 | 149 (3.5%) | 39 (1.2%) | 110 (10.1%) |  |
| 4 | 90 (2.1%) | 17 (0.5%) | 73 (6.7%) |  |
| **Albumin (g/dL)** | 3.48 ± 0.67 | 3.61 ± 0.60 | 3.09 ± 0.72 | < 0.001 |
| **Total bilirubin (mg/dL)** | 2.56 ± 4.30 | 2.13 ± 3.67 | 3.80 ± 5.58 | < 0.001 |
| **INR** | 1.22 ± 0.93 | 1.19 ± 1.05 | 1.31 ± 0.40 | < 0.001 |
| **Creatinine (mg/dL)** | 0.97 ± 0.66 | 0.94 ± 0.59 | 1.05 ± 0.82 | < 0.001 |
| **Sodium (mmol/L)** | 136.61 ± 4.63 | 137.18 ± 4.24 | 134.93 ± 5.26 | < 0.001 |
| **ALT (IU/mL)** | 74.60 ± 151.77 | 72.92 ± 162.81 | 79.53 ± 113.28 | 0.043 |
| **Platelet (10^3^/uL)** | 191.87 ± 110.20 | 190.61 ± 104.25 | 195.58 ± 126.00 | 0.050 |
| **AFP (ng/mL)** | 35,041.52 ± 157,429.50 | 32,946.44 ± 143,112.77 | 41,182.60 ± 193,328.98 | 0.793 |
| **Multiple tumors** | 2,617 (61.1%) | 1,872 (58.6%) | 745 (68.3%) | < 0.001 |
| **Maximum tumor diameter (cm)** | 7.00 ± 4.64 | 6.86 ± 4.53 | 7.43 ± 4.93 | 0.018 |
| **Portal vein invasion** | 3,119 (72.8%) | 2,336 (73.1%) | 783 (71.8%) | 0.431 |
| **Hepatic vein invasion** | 747 (17.4%) | 541 (16.9%) | 206 (18.9%) | 0.139 |
| **Bile duct invasion** | 339 (7.9%) | 262 (8.2%) | 77 (7.1%) | 0.243 |
| **Hepatic artery invasion** | 141 (3.3%) | 104 (3.3%) | 37 (3.4%) | 0.844 |
| **Lymph node metastasis** | 921 (21.5%) | 646 (20.2%) | 275 (25.2%) | 0.001 |
| **Extrahepatic metastasis** | 1,408 (32.9%) | 941 (29.5%) | 467 (42.8%) | < 0.001 |
| **Ascites** |  |  |  | < 0.001 |
| None | 2,192 (51.2%) | 1,837 (57.5%) | 355 (32.6%) |  |
| Mild | 1,188 (27.7%) | 841 (26.3%) | 347 (31.8%) |  |
| Moderate to severe | 905 (21.1%) | 517 (16.2%) | 388 (35.6%) |  |
| **Hepatic encephalopathy grade** |  |  |  | < 0.001 |
| None | 4,127 (96.3%) | 3,093 (96.8%) | 1,034 (94.9%) |  |
| Grade 1 or 2 | 120 (2.8%) | 83 (2.6%) | 37 (3.4%) |  |
| Grade 3 or 4 | 38 (0.9%) | 19 (0.6%) | 19 (1.7%) |  |
| **Child-Pugh classification** |  |  |  | < 0.001 |
| A | 2,981 (69.6%) | 2,457 (76.9%) | 524 (48.1%) |  |
| B | 1,200 (28.0%) | 706 (22.1%) | 494 (45.3%) |  |
| C | 104 (2.4%) | 32 (1.0%) | 72 (6.6%) |  |
| **MELD score** | 11.21 ± 5.02 | 10.47 ± 4.31 | 13.38 ± 6.19 | < 0.001 |

^1^n (%); Mean ± SD, ^2^Fisher’s exact test

ECOG, Eastern Cooperative Oncology Group; INR, international normalized ratio; ALT, Alanine aminotransferase; AFP, alpha-fetoprotein; BCLC, Barcelona clinic liver cancer; MELD, model for end-stage liver disease.
