# Supplementary material for: Evaluating the clinical utility of large language models for hepatocellular carcinoma treatment recommendations: A nationwide retrospective registry study
Source: PLoS Med. 2026 Jan 13;23(1):e1004855. doi: 10.1371/journal.pmed.1004855 (PMC12799000; doi:10.1371/journal.pmed.1004855)
Supplement: S12 Table — (DOCX) [file pmed.1004855.s026.docx]

**S12 Table. Baseline clinical characteristics according to concordance between physician decisions and Claude 3.5-generated treatment recommendations in BCLC stage A.**

| **Clinical characteristics** | **Overall (n^1^ = 4,064)** | **Treatment concordance with Claude** | | ***P* value^2^** |
| --- | --- | --- | --- | --- |
|  |  | **Mismatch (n^1^ = 3,006)** | **Match (n^1^ = 1,058)** |  |
| **Age at diagnosis** | 62.56 ± 10.56 | 62.87 ± 10.65 | 61.67 ± 10.27 | 0.593 |
| **Sex** |  |  |  | 0.014 |
| Male | 2,967 (73.0%) | 2,164 (72.0%) | 803 (75.9%) |  |
| Female | 1,097 (27.0%) | 842 (28.0%) | 255 (24.1%) |  |
| **Diabetes mellitus** | 1,232 (30.3%) | 915 (30.4%) | 317 (30.0%) | 0.786 |
| **Hypertension** | 1,497 (36.8%) | 1,113 (37.0%) | 384 (36.3%) | 0.684 |
| **Hepatitis B** | 2,265 (55.7%) | 1,653 (55.0%) | 612 (57.8%) | 0.113 |
| **Hepatitis C** | 546 (13.4%) | 422 (14.0%) | 124 (11.7%) | 0.059 |
| **Past smoking history** | 1,635 (40.2%) | 1,214 (40.4%) | 421 (39.8%) | 0.743 |
| **Past alcohol use** | 1,307 (32.2%) | 953 (31.7%) | 354 (33.5%) | 0.302 |
| **Albumin (g/dL)** | 3.86 ± 0.65 | 3.82 ± 0.65 | 3.98 ± 0.66 | < 0.001 |
| **Total bilirubin (mg/dL)** | 1.30 ± 1.87 | 1.26 ± 1.45 | 1.40 ± 2.73 | 0.169 |
| **INR** | 1.15 ± 0.21 | 1.15 ± 0.19 | 1.14 ± 0.24 | < 0.001 |
| **Creatinine (mg/dL)** | 0.97 ± 0.87 | 0.98 ± 0.95 | 0.94 ± 0.58 | 0.127 |
| **Sodium (mmol/L)** | 139.08 ± 5.44 | 138.99 ± 5.20 | 139.35 ± 6.07 | 0.774 |
| **ALT (IU/mL)** | 40.12 ± 52.34 | 39.89 ± 52.23 | 40.75 ± 52.66 | 0.133 |
| **Platelet (10^3^/uL)** | 132.57 ± 64.82 | 129.40 ± 65.92 | 141.59 ± 60.70 | 0.017 |
| **AFP (ng/mL)** | 1,741.52 ± 83,226.43 | 2,265.42 ± 96,751.17 | 252.98 ± 3,165.34 | 0.923 |
| **Maximum tumor diameter (cm)** | 1.91 ± 0.63 | 1.91 ± 0.64 | 1.93 ± 0.61 | 0.321 |
| **MELD score** | 9.38 ± 3.55 | 9.45 ± 3.39 | 9.19 ± 3.98 | < 0.001 |

^1^n (%); Mean ± SD, ^2^Fisher’s exact test

INR, international normalized ratio; ALT, Alanine aminotransferase; AFP, alpha-fetoprotein; MELD, model for end-stage liver disease.
