# Supplementary material for: Evaluating the clinical utility of large language models for hepatocellular carcinoma treatment recommendations: A nationwide retrospective registry study
Source: PLoS Med. 2026 Jan 13;23(1):e1004855. doi: 10.1371/journal.pmed.1004855 (PMC12799000; doi:10.1371/journal.pmed.1004855)
Supplement: S13 Table — (DOCX) [file pmed.1004855.s027.docx]

**S13 Table. Baseline clinical characteristics according to concordance between physician decisions and Claude 3.5-generated treatment recommendations in BCLC stage C.**

| **Clinical characteristics** | **Overall (n^1^ = 4,285)** | **Treatment concordance with Claude** | | ***P* value^2^** |
| --- | --- | --- | --- | --- |
|  |  | **Mismatch (n^1^ = 3,443)** | **Match (n^1^ = 842)** |  |
| **Age at diagnosis** | 60.59 ± 12.17 | 60.48 ± 12.04 | 61.04 ± 12.68 | 0.307 |
| **Sex** |  |  |  | 0.267 |
| Male | 3,614 (84.3%) | 2,893 (84.0%) | 721 (85.6%) |  |
| Female | 671 (15.7%) | 550 (16.0%) | 121 (14.4%) |  |
| **Diabetes mellitus** | 1,057 (24.7%) | 832 (24.2%) | 225 (26.7%) | 0.129 |
| **Hypertension** | 1,389 (32.4%) | 1,120 (32.5%) | 269 (31.9%) | 0.774 |
| **Hepatitis B** | 2,602 (60.7%) | 2,099 (61.0%) | 503 (59.7%) | 0.529 |
| **Hepatitis C** | 396 (9.2%) | 313 (9.1%) | 83 (9.9%) | 0.507 |
| **Past smoking history** | 2,228 (52.0%) | 1,772 (51.5%) | 456 (54.2%) | 0.166 |
| **Past alcohol use** | 1,779 (41.5%) | 1,410 (41.0%) | 369 (43.8%) | 0.138 |
| **ECOG performance status** |  |  |  | < 0.001 |
| 0 | 1,540 (35.9%) | 1,319 (38.3%) | 221 (26.2%) |  |
| 1 | 1,566 (36.5%) | 1,296 (37.6%) | 270 (32.1%) |  |
| 2 | 940 (21.9%) | 772 (22.4%) | 168 (20.0%) |  |
| 3 | 149 (3.5%) | 39 (1.1%) | 110 (13.1%) |  |
| 4 | 90 (2.1%) | 17 (0.5%) | 73 (8.7%) |  |
| **Albumin (g/dL)** | 3.48 ± 0.67 | 3.54 ± 0.66 | 3.23 ± 0.69 | < 0.001 |
| **Total bilirubin (mg/dL)** | 2.56 ± 4.30 | 1.89 ± 2.69 | 5.30 ± 7.43 | < 0.001 |
| **INR** | 1.22 ± 0.93 | 1.19 ± 1.01 | 1.34 ± 0.46 | < 0.001 |
| **Creatinine (mg/dL)** | 0.97 ± 0.66 | 0.92 ± 0.49 | 1.18 ± 1.09 | < 0.001 |
| **Sodium (mmol/L)** | 136.61 ± 4.63 | 137.03 ± 4.35 | 134.87 ± 5.27 | < 0.001 |
| **ALT (IU/mL)** | 74.60 ± 151.77 | 71.43 ± 157.05 | 87.56 ± 127.18 | 0.002 |
| **Platelet (10^3^/uL)** | 191.87 ± 110.20 | 189.88 ± 104.82 | 200.02 ± 129.64 | 0.049 |
| **AFP (ng/mL)** | 35,041.52 ± 157,429.50 | 32,588.64 ± 147,472.28 | 45,071.53 ± 192,648.98 | 0.514 |
| **Multiple tumors** | 2,617 (61.1%) | 2,046 (59.4%) | 571 (67.8%) | < 0.001 |
| **Maximum tumor diameter (cm)** | 7.00 ± 4.64 | 6.89 ± 4.52 | 7.46 ± 5.08 | 0.001 |
| **Portal vein invasion** | 3,119 (72.8%) | 2,506 (72.8%) | 613 (72.8%) | 1.000 |
| **Hepatic vein invasion** | 747 (17.4%) | 576 (16.7%) | 171 (20.3%) | 0.015 |
| **Bile duct invasion** | 339 (7.9%) | 263 (7.6%) | 76 (9.0%) | 0.200 |
| **Hepatic artery invasion** | 141 (3.3%) | 115 (3.3%) | 26 (3.1%) | 0.829 |
| **Lymph node metastasis** | 921 (21.5%) | 722 (21.0%) | 199 (23.6%) | 0.092 |
| **Extrahepatic metastasis** | 1,408 (32.9%) | 1,046 (30.4%) | 362 (43.0%) | < 0.001 |
| **Ascites** |  |  |  | < 0.001 |
| None | 2,192 (51.2%) | 1,907 (55.4%) | 285 (33.8%) |  |
| Mild | 1,188 (27.7%) | 919 (26.7%) | 269 (31.9%) |  |
| Moderate to severe | 905 (21.1%) | 617 (17.9%) | 288 (34.2%) |  |
| **Hepatic encephalopathy grade** |  |  |  | < 0.001 |
| None | 4,127 (96.3%) | 3,340 (97.0%) | 787 (93.5%) |  |
| Grade 1 or 2 | 120 (2.8%) | 84 (2.4%) | 36 (4.3%) |  |
| Grade 3 or 4 | 38 (0.9%) | 19 (0.6%) | 19 (2.3%) |  |
| **Child-Pugh classification** |  |  |  | < 0.001 |
| A | 2,981 (69.6%) | 2,553 (74.2%) | 428 (50.8%) |  |
| B | 1,200 (28.0%) | 859 (24.9%) | 341 (40.5%) |  |
| C | 104 (2.4%) | 31 (0.9%) | 73 (8.7%) |  |
| **MELD score** | 11.21 ± 5.02 | 10.36 ± 3.90 | 14.71 ± 7.14 | < 0.001 |

^1^n (%); Mean ± SD, ^2^Fisher’s exact test

ECOG, Eastern Cooperative Oncology Group; INR, international normalized ratio; ALT, Alanine aminotransferase; AFP, alpha-fetoprotein; BCLC, Barcelona clinic liver cancer; MELD, model for end-stage liver disease.
