# Supplementary material for: Evaluating the clinical utility of large language models for hepatocellular carcinoma treatment recommendations: A nationwide retrospective registry study
Source: PLoS Med. 2026 Jan 13;23(1):e1004855. doi: 10.1371/journal.pmed.1004855 (PMC12799000; doi:10.1371/journal.pmed.1004855)
Supplement: S14 Table — (DOCX) [file pmed.1004855.s028.docx]

**S14 Table. Top 10 most frequent disagreement patterns between LLM recommendations and actual treatments in the entire cohort.**

| **ChatGPT 4o** | | | |
| --- | --- | --- | --- |
| **Rank** | **Actual treatment** | **ChatGPT recommendation** | **Count** |
| 1 | Surgical resection | Conventional TACE | 1,323 |
| 2 | Conventional TACE | Surgical resection | 843 |
| 3 | RFA | Surgical resection | 839 |
| 4 | Best supportive care | Sorafenib | 670 |
| 5 | Best supportive care | Conventional TACE | 658 |
| 6 | Conventional TACE | Sorafenib | 511 |
| 7 | Conventional TACE | Best supportive care | 503 |
| 8 | Sorafenib | Best supportive care | 326 |
| 9 | RFA | Conventional TACE | 262 |
| 10 | Conventional TACE | RFA | 234 |
| **Gemini 2.0** | | | |
| **Rank** | **Actual treatment** | **Gemini recommendation** | **Count** |
| 1 | Surgical resection | Conventional TACE | 1,268 |
| 2 | Best supportive care | Sorafenib | 939 |
| 3 | Conventional TACE | Sorafenib | 810 |
| 4 | Conventional TACE | RFA | 606 |
| 5 | Conventional TACE | Best supportive care | 532 |
| 6 | Best supportive care | Conventional TACE | 467 |
| 7 | Surgical resection | RFA | 465 |
| 8 | Conventional TACE | Surgical resection | 419 |
| 9 | RFA | Surgical resection | 249 |
| 10 | RFA | Conventional TACE | 227 |
| **Claude 3.5** | | | |
| **Rank** | **Actual treatment** | **Claude recommendation** | **Count** |
| 1 | Surgical resection | Conventional TACE | 1,072 |
| 2 | Best supportive care | Sorafenib | 999 |
| 3 | Conventional TACE | Sorafenib | 824 |
| 4 | Conventional TACE | RFA | 576 |
| 5 | Surgical resection | RFA | 530 |
| 6 | Conventional TACE | Surgical resection | 526 |
| 7 | Best supportive care | Conventional TACE | 488 |
| 8 | RFA | Surgical resection | 460 |
| 9 | Conventional TACE | TACE with beads | 374 |
| 10 | Surgical resection | TACE with beads | 240 |

LLM, large language model; TACE, transarterial chemoembolization; RFA, radiofrequency ablation.
