# Supplementary material for: Evaluating the clinical utility of large language models for hepatocellular carcinoma treatment recommendations: A nationwide retrospective registry study
Source: PLoS Med. 2026 Jan 13;23(1):e1004855. doi: 10.1371/journal.pmed.1004855 (PMC12799000; doi:10.1371/journal.pmed.1004855)
Supplement: S15 Table — (DOCX) [file pmed.1004855.s029.docx]

**S15 Table. Median overall survival according to concordance between LLM recommendations and actual treatment across BCLC stages.**

| **Median overall survival (months, 95% CI) in BCLC stage A** | | | | |
| --- | --- | --- | --- | --- |
| **Actual treatment** | **Overall patients** | **Concordant with ChatGPT 4o recommendation** | **Concordant with Gemini 2.0 recommendation** | **Concordant with Claude 3.5 recommendation** |
| Conventional TACE | 49.1 (47.5-51.7) | NA | NA | 36.9 (9.2-56.0) |
| Surgical resection | 69.5 (64.4-74.1) | 70.0 (65.3-75.1) | 69.0 (62.2-74.1) | 72.3 (67.0-77.7) |
| RFA | 58.4 (54.5-62.2) | 42.9 (30.8-45.6) | 63.9 (58.4-67.9) | 57.3 (52.7-63.9) |
| Best supportive care | 11.7 (8.8-15.2) | 5.5 (3.5-7.5) | 6.2 (5.1-8.2) | 4.6 (2.1-7.3) |
| **Median overall survival (months, 95% CI) in BCLC stage C** | | | | |
| **Actual treatment** | **Overall patients** | **Concordant with ChatGPT 4o recommendation** | **Concordant with Gemini 2.0 recommendation** | **Concordant with Claude 3.5 recommendation** |
| Best supportive care | 2.1 (2.0-2.2) | 2.0 (1.9-2.1) | 1.7 (1.5-1.8) | 1.4(1.3-1.6) |
| Conventional TACE | 10.3 (9.4-11.8) | NA | NA | 7.7 (4.0-NA) |
| Sorafenib | 4.1(3.9-4.4) | 4.1 (3.9-4.7) | 4.1(3.9-4.6) | 3.9 (3.4-4.1) |
| Surgical resection | 26.6 (18.5-32.8) | NA | NA | NA |
| Radiation therapy | 5.2 (4.6-6.2) | NA | NA | NA |

TACE, transarterial chemoembolization; RFA, radiofrequency ablation; OS, overall survival; CI, confidence interval; NA, not applicable.
