# Supplementary material for: Evaluating the clinical utility of large language models for hepatocellular carcinoma treatment recommendations: A nationwide retrospective registry study
Source: PLoS Med. 2026 Jan 13;23(1):e1004855. doi: 10.1371/journal.pmed.1004855 (PMC12799000; doi:10.1371/journal.pmed.1004855)
Supplement: S16 Table — (DOCX) [file pmed.1004855.s030.docx]

**S16 Table. Stage-specific adherence and treatment-tier transition patterns across large language models.**

| **BCLC stage** | **LLM** | **Higher-tier recommendation (%)** | **Same-tier recommendation (%)** | **Lower-tier recommendation (%)** |
| --- | --- | --- | --- | --- |
| **A** | **ChatGPT 4o** | 39.5 | 56.4 | 4.1 |
|  | **Gemini 2.0** | 35.5 | 56.9 | 7.6 |
|  | **Claude 3.5** | 39.6 | 57.1 | 3.2 |
| **B** | **ChatGPT 4o** | 15.7 | 49.9 | 34.3 |
|  | **Gemini 2.0** | 11.7 | 48.8 | 39.5 |
|  | **Claude 3.5** | 14.1 | 50.0 | 36.0 |
| **C** | **ChatGPT 4o** | 22.0 | 28.5 | 49.5 |
|  | **Gemini 2.0** | 27.1 | 30.4 | 42.5 |
|  | **Claude 3.5** | 32.4 | 26.3 | 41.3 |

Treatment tiers were ordered as follows: (1) curative-intent (resection, transplantation, RFA), (2) locoregional non-curative (TACE, radioembolization), (3) systemic therapy, and (4) best supportive care. LLM, large language model; BCLC, Barcelona clinic liver cancer.
