# Supplementary material for: Evaluating the clinical utility of large language models for hepatocellular carcinoma treatment recommendations: A nationwide retrospective registry study
Source: PLoS Med. 2026 Jan 13;23(1):e1004855. doi: 10.1371/journal.pmed.1004855 (PMC12799000; doi:10.1371/journal.pmed.1004855)
Supplement: S17 Table — (DOCX) [file pmed.1004855.s031.docx]

**S17 Table. Within-stage IPTW-adjusted effects of tier escalation on overall survival across large language models.**

| **BCLC stage** | **LLM** | **HR** | **95% CI** | ***P* value** |
| --- | --- | --- | --- | --- |
| **A** | **ChatGPT 4o** | 0.67 | 0.60-1.66 | < 0.001 |
|  | **Gemini 2.0** | 0.68 | 0.62-1.62 | < 0.001 |
|  | **Claude 3.5** | 0.71 | 0.64-1.55 | < 0.001 |
| **B** | **ChatGPT 4o** | 0.41 | 0.36-2.76 | < 0.001 |
|  | **Gemini 2.0** | 0.43 | 0.38-2.65 | < 0.001 |
|  | **Claude 3.5** | 0.44 | 0.39-2.58 | < 0.001 |
| **C** | **ChatGPT 4o** | 0.40 | 0.35-2.83 | < 0.001 |
|  | **Gemini 2.0** | 0.46 | 0.42-2.36 | < 0.001 |
|  | **Claude 3.5** | 0.48 | 0.44-2.28 | < 0.001 |

Treatment tiers were ordered as follows: (1) curative-intent (resection, transplantation, RFA), (2) locoregional non-curative (TACE, radioembolization), (3) systemic therapy, and (4) best supportive care. Tier escalation was defined as an LLM recommending a higher-tier therapy relative to the actual treatment received. LLM, large language model; BCLC, Barcelona clinic liver cancer; HR, hazard ratios; CI, CI, confidence interval. *P* values were calculated from IPTW-adjusted Cox proportional hazards models.
