# Supplementary material for: Evaluating the clinical utility of large language models for hepatocellular carcinoma treatment recommendations: A nationwide retrospective registry study
Source: PLoS Med. 2026 Jan 13;23(1):e1004855. doi: 10.1371/journal.pmed.1004855 (PMC12799000; doi:10.1371/journal.pmed.1004855)
Supplement: S18 Table — (DOCX) [file pmed.1004855.s032.docx]

**S18 Table. Baseline clinical characteristics of HCC patients according to the LLM model before IPTW.**

| **Clinical characteristics** | **Overall**  **(n^1^ = 6,078)** | **Large language model** | | | **Maximum SMD^2^** |
| --- | --- | --- | --- | --- | --- |
|  |  | **ChatGPT 4o**  **(n^1^ = 2,190)** | **Gemini 2.0**  **(n^1^ = 2,297)** | **Claude 3.5**  **(n^1^ = 1,591)** |  |
| **Age at diagnosis** | 62.86 ± 11.48 | 62.36 ± 11.45 | 63.42 ± 11.53 | 62.74 ± 11.43 | 0.093 |
| **Sex** |  |  |  |  | 0.023 |
| Male | 4,806 (79.1%) | 1,734 (79.2%) | 1,806 (78.6%) | 1,266 (79.6%) |  |
| Female | 1,272 (20.9%) | 456 (20.8%) | 491 (21.4%) | 325 (20.4%) |  |
| **Diabetes mellitus** | 1,803 (29.7%) | 620 (28.3%) | 699 (30.4%) | 484 (30.4%) | 0.047 |
| **Hypertension** | 2,314 (38.1%) | 799 (36.5%) | 914 (39.8%) | 601 (37.8%) | 0.068 |
| **Hepatitis B** | 3,388 (55.7%) | 1,278 (58.4%) | 1,243 (54.1%) | 867 (54.5%) | 0.086 |
| **Hepatitis C** | 782 (12.9%) | 241 (11.0%) | 332 (14.5%) | 209 (13.1%) | 0.104 |
| **Past smoking history** | 2,781 (45.8%) | 1,011 (46.2%) | 1,046 (45.5%) | 724 (45.5%) | 0.013 |
| **Past alcohol use** | 2,249 (37.0%) | 796 (36.3%) | 847 (36.9%) | 606 (38.1%) | 0.036 |
| **ECOG performance status** |  |  |  |  | 0.077 |
| 0 | 2,941 (48.4%) | 1,088 (49.7%) | 1,095 (47.7%) | 758 (47.6%) |  |
| 1 | 1,780 (29.3%) | 630 (28.8%) | 674 (29.3%) | 476 (29.9%) |  |
| 2 | 1,077 (17.7%) | 402 (18.4%) | 400 (17.4%) | 275 (17.3%) |  |
| 3 | 170 (2.8%) | 48 (2.2%) | 72 (3.1%) | 50 (3.1%) |  |
| 4 | 110 (1.8%) | 22 (1.0%) | 56 (2.4%) | 32 (2.0%) |  |
| **Albumin (g/dL)** | 3.67 ± 0.71 | 3.72 ± 0.68 | 3.63 ± 0.75 | 3.64 ± 0.70 | 0.139 |
| **Total bilirubin (mg/dL)** | 1.93 ± 3.64 | 1.65 ± 2.85 | 1.76 ± 3.04 | 2.56 ± 5.07 | 0.220 |
| **INR** | 1.17 ± 0.28 | 1.16 ± 0.23 | 1.17 ± 0.27 | 1.20 ± 0.34 | 0.138 |
| **Creatinine (mg/dL)** | 1.01 ± 0.88 | 0.99 ± 0.90 | 0.99 ± 0.81 | 1.06 ± 0.96 | 0.073 |
| **Sodium (mmol/L)** | 137.84 ± 5.72 | 137.88 ± 6.28 | 137.82 ± 5.61 | 137.81 ± 5.01 | 0.013 |
| **ALT (IU/mL)** | 55.69 ± 82.77 | 57.61 ± 93.45 | 52.98 ± 77.81 | 56.96 ± 73.50 | 0.054 |
| **Platelet (10^3^/uL)** | 163.19 ± 93.11 | 168.89 ± 96.05 | 161.56 ± 90.68 | 157.71 ± 92.08 | 0.119 |
| **AFP (ng/mL)** | 14,842.21 ± 115,809.67 | 15,308.05 ± 119,657.56 | 14,733.08 ± 133,567.14 | 14,358.52 ± 76,067.28 | 0.009 |
| **Multiple tumors** | 2,699 (44.4%) | 1,002 (45.8%) | 991 (43.1%) | 706 (44.4%) | 0.053 |
| **Maximum tumor diameter (cm)** | 4.53 ± 3.98 | 4.72 ± 4.07 | 4.45 ± 3.94 | 4.39 ± 3.90 | 0.082 |
| **Portal vein invasion** | 1,217 (20.0%) | 394 (18.0%) | 510 (22.2%) | 313 (19.7%) | 0.105 |
| **Hepatic vein invasion** | 350 (5.8%) | 152 (6.9%) | 122 (5.3%) | 76 (4.8%) | 0.092 |
| **Bile duct invasion** | 161 (2.6%) | 63 (2.9%) | 58 (2.5%) | 40 (2.5%) | 0.022 |
| **Hepatic artery invasion** | 68 (1.1%) | 31 (1.4%) | 27 (1.2%) | 10 (0.6%) | 0.078 |
| **Lymph node metastasis** | 520 (8.6%) | 252 (11.5%) | 160 (7.0%) | 108 (6.8%) | 0.164 |
| **Extrahepatic metastasis** | 914 (15.0%) | 491 (22.4%) | 249 (10.8%) | 174 (10.9%) | 0.315 |
| **Ascites** |  |  |  |  | 0.032 |
| None | 4,356 (71.7%) | 1,582 (72.2%) | 1,647 (71.7%) | 1,127 (70.8%) |  |
| Mild | 998 (16.4%) | 365 (16.7%) | 357 (15.5%) | 276 (17.3%) |  |
| Moderate to severe | 724 (11.9%) | 243 (11.1%) | 293 (12.8%) | 188 (11.8%) |  |
| **Hepatic encephalopathy grade** |  |  |  |  | 0.029 |
| None | 5,921 (97.4%) | 2,136 (97.5%) | 2,240 (97.5%) | 1,545 (97.1%) |  |
| Grade 1 or 2 | 125 (2.1%) | 40 (1.8%) | 48 (2.1%) | 37 (2.3%) |  |
| Grade 3 or 4 | 32 (0.5%) | 14 (0.6%) | 9 (0.4%) | 9 (0.6%) |  |
| **Child-Pugh classification** |  |  |  |  | 0.126 |
| A | 4,780 (78.6%) | 1,778 (81.2%) | 1,764 (76.8%) | 1,238 (77.8%) |  |
| B | 1,200 (19.7%) | 387 (17.7%) | 503 (21.9%) | 310 (19.5%) |  |
| C | 98 (1.6%) | 25 (1.1%) | 30 (1.3%) | 43 (2.7%) |  |
| **BCLC stage** |  |  |  |  | 0.147 |
| A | 2,065 (34.0%) | 679 (31.0%) | 843 (36.7%) | 543 (34.1%) |  |
| B | 2,318 (38.1%) | 826 (37.7%) | 837 (36.4%) | 655 (41.2%) |  |
| C | 1,695 (27.9%) | 685 (31.3%) | 617 (26.9%) | 393 (24.7%) |  |
| **MELD score** | 10.25 ± 4.65 | 9.92 ± 4.14 | 10.09 ± 4.28 | 10.94 ± 5.64 | 0.207 |

^1^n (%); Mean ± SD, ^2^The maximum SMD among all pairwise comparisons between the three LLM groups was reported for each variable.

HCC, hepatocellular carcinoma; LLM, large language model; IPTW, inverse probability of treatment weighting; SMD, standardized mean differences; ECOG, Eastern Cooperative Oncology Group; INR, international normalized ratio; ALT, Alanine aminotransferase; AFP, alpha-fetoprotein; BCLC, Barcelona clinic liver cancer; MELD, model for end-stage liver disease.
