# Supplementary material for: Evaluating the clinical utility of large language models for hepatocellular carcinoma treatment recommendations: A nationwide retrospective registry study
Source: PLoS Med. 2026 Jan 13;23(1):e1004855. doi: 10.1371/journal.pmed.1004855 (PMC12799000; doi:10.1371/journal.pmed.1004855)
Supplement: S19 Table — (DOCX) [file pmed.1004855.s033.docx]

**S19 Table. Baseline clinical characteristics of HCC patients according to the LLM model after IPTW.**

| **Clinical characteristics** | **Overall**  **(n^1^ = 5,941)** | **Large language model** | | | **Maximum SMD^2^** |
| --- | --- | --- | --- | --- | --- |
|  |  | **ChatGPT 4o**  **(n^1^ = 2,124)** | **Gemini 2.0**  **(n^1^ = 2,253)** | **Claude 3.5**  **(n^1^ = 1,564)** |  |
| **Age at diagnosis** | 62.78 ± 11.45 | 62.85 ± 11.38 | 62.81 ± 11.49 | 62.63 ± 11.49 | 0.013 |
| **Sex** |  |  |  |  | 0.004 |
| Male | 4,819 (79.5%) | 1,716 (79.6%) | 1,837 (79.7%) | 1,267 (79.4%) |  |
| Female | 1,239 (20.5%) | 441 (20.4%) | 469 (20.3%) | 329 (20.6%) |  |
| **Diabetes mellitus** | 1,797 (29.7%) | 638 (29.6%) | 687 (29.8%) | 472 (29.6%) | 0.004 |
| **Hypertension** | 2,304 (38.0%) | 820 (38.0%) | 877 (38.0%) | 608 (38.1%) | 0.001 |
| **Hepatitis B** | 3,397 (56.1%) | 1,209 (56.1%) | 1,290 (55.9%) | 898 (56.3%) | 0.004 |
| **Hepatitis C** | 763 (12.6%) | 268 (12.4%) | 295 (12.8%) | 200 (12.6%) | 0.007 |
| **Past smoking history** | 2,789 (46.0%) | 988 (45.8%) | 1,060 (46.0%) | 741 (46.5%) | 0.009 |
| **Past alcohol use** | 2,271 (37.5%) | 807 (37.4%) | 863 (37.4%) | 602 (37.7%) | 0.004 |
| **ECOG performance status** |  |  |  |  | 0.027 |
| 0 | 2,957 (48.8%) | 1,053 (48.9%) | 1,129 (48.9%) | 775 (48.5%) |  |
| 1 | 1,757 (29.0%) | 629 (29.2%) | 665 (28.8%) | 463 (29.0%) |  |
| 2 | 1,048 (17.3%) | 394 (18.3%) | 388 (16.8%) | 267 (16.7%) |  |
| 3 | 178 (2.9%) | 54 (2.5%) | 68 (3.0%) | 55 (3.4%) |  |
| 4 | 119 (2.0%) | 26 (1.2%) | 57 (2.5%) | 36 (2.3%) |  |
| **Albumin (g/dL)** | 3.67 ± 0.71 | 3.68 ± 0.69 | 3.67 ± 0.74 | 3.67 ± 0.70 | 0.018 |
| **Total bilirubin (mg/dL)** | 1.90 ± 3.65 | 1.79 ± 3.41 | 1.94 ± 3.70 | 1.97 ± 3.87 | 0.033 |
| **INR** | 1.17 ± 0.27 | 1.17 ± 0.24 | 1.17 ± 0.27 | 1.18 ± 0.29 | 0.018 |
| **Creatinine (mg/dL)** | 1.02 ± 0.90 | 1.01 ± 0.93 | 1.02 ± 0.88 | 1.01 ± 0.88 | 0.014 |
| **Sodium (mmol/L)** | 137.87 ± 5.60 | 137.92 ± 6.10 | 137.80 ± 5.62 | 137.91 ± 4.82 | 0.015 |
| **ALT (IU/mL)** | 55.32 ± 82.71 | 54.84 ± 82.97 | 55.37 ± 87.73 | 55.88 ± 74.51 | 0.009 |
| **Platelet (10^3^/uL)** | 163.30 ± 92.77 | 163.19 ± 91.10 | 163.17 ± 92.26 | 163.63 ± 95.77 | 0.003 |
| **AFP (ng/mL)** | 14,417.21 ± 114,999.66 | 14,189.10 ± 122,719.13 | 14,440.16 ± 129,088.28 | 14,692.18 ± 76,112.43 | 0.003 |
| **Multiple tumors** | 2,690 (44.4%) | 950 (44.1%) | 1,026 (44.5%) | 713 (44.7%) | 0.009 |
| **Maximum tumor diameter (cm)** | 4.53 ± 3.98 | 4.49 ± 3.91 | 4.53 ± 3.99 | 4.56 ± 4.07 | 0.010 |
| **Portal vein invasion** | 1,151 (19.0%) | 396 (18.3%) | 445 (19.3%) | 310 (19.4%) | 0.018 |
| **Hepatic vein invasion** | 358 (5.9%) | 124 (5.8%) | 142 (6.2%) | 92 (5.7%) | 0.012 |
| **Bile duct invasion** | 155 (2.6%) | 53 (2.4%) | 58 (2.5%) | 43 (2.7%) | 0.012 |
| **Hepatic artery invasion** | 64 (1.1%) | 22 (1.0%) | 26 (1.1%) | 16 (1.0%) | 0.011 |
| **Lymph node metastasis** | 526 (8.7%) | 186 (8.6%) | 205 (8.9%) | 135 (8.5%) | 0.009 |
| **Extrahepatic metastasis** | 954 (15.7%) | 342 (15.9%) | 362 (15.7%) | 250 (15.6%) | 0.004 |
| **Ascites** |  |  |  |  | 0.012 |
| None | 4,390 (72.5%) | 1,566 (72.6%) | 1,676 (72.7%) | 1,148 (71.9%) |  |
| Mild | 985 (16.3%) | 360 (16.7%) | 355 (15.4%) | 270 (16.9%) |  |
| Moderate to severe | 684 (11.3%) | 231 (10.7%) | 275 (11.9%) | 178 (11.1%) |  |
| **Hepatic encephalopathy grade** |  |  |  |  | 0.006 |
| None | 5,907 (97.5%) | 2,102 (97.5%) | 2,248 (97.5%) | 1,557 (97.5%) |  |
| Grade 1 or 2 | 120 (2.0%) | 41 (1.9%) | 48 (2.1%) | 32 (2.0%) |  |
| Grade 3 or 4 | 31 (0.5%) | 13 (0.6%) | 11 (0.5%) | 8 (0.5%) |  |
| **Child-Pugh classification** |  |  |  |  | 0.026 |
| A | 4,819 (79.5%) | 1,733 (80.4%) | 1,820 (78.9%) | 1,265 (79.2%) |  |
| B | 1,139 (18.8%) | 390 (18.1%) | 446 (19.4%) | 303 (19.0%) |  |
| C | 101 (1.7%) | 33 (1.5%) | 39 (1.7%) | 28 (1.8%) |  |
| **BCLC stage** |  |  |  |  | 0.021 |
| A | 2,059 (34.0%) | 741 (34.4%) | 783 (34.0%) | 535 (33.5%) |  |
| B | 2,334 (38.5%) | 838 (38.9%) | 881 (38.2%) | 616 (38.6%) |  |
| C | 1,665 (27.5%) | 577 (26.8%) | 642 (27.8%) | 446 (28.0%) |  |
| **MELD score** | 10.20 ± 4.65 | 10.08 ± 4.37 | 10.27 ± 4.73 | 10.25 ± 4.88 | 0.028 |

^1^n (%); Mean ± SD, ^2^The maximum SMD among all pairwise comparisons between the three LLM groups was reported for each variable.

HCC, hepatocellular carcinoma; LLM, large language model; IPTW, inverse probability of treatment weighting; SMD, standardized mean differences; ECOG, Eastern Cooperative Oncology Group; INR, international normalized ratio; AFP, alpha-fetoprotein; BCLC, Barcelona clinic liver cancer; MELD, model for end-stage liver disease.
