# Supplementary material for: Evaluating the clinical utility of large language models for hepatocellular carcinoma treatment recommendations: A nationwide retrospective registry study
Source: PLoS Med. 2026 Jan 13;23(1):e1004855. doi: 10.1371/journal.pmed.1004855 (PMC12799000; doi:10.1371/journal.pmed.1004855)
Supplement: Attachment — Submitted filename: Response letter_1st revision.docx [file pmed.1004855.s037.docx]

[Comments from the senior editor]

Many thanks for submitting your manuscript "Evaluating the Clinical Utility of Large Language Models for Hepatocellular Carcinoma Treatment Recommendations" (PMEDICINE-D-25-03475R1) to PLOS Medicine. The paper has been reviewed by subject experts, a statistician and an academic editor; their comments are included below and can also be accessed here:

As you will see, while the reviewers find the work interesting, they raise important concerns and identify the need for additional analyses to consider the study further. In particular the reviewers require additional information pertaining to the prompts used, the method of determining concordance between physician and LLM, consideration of potential bias associated with use of overall survival as outcome, and that you address discordance between physician recommendation and actual treatment. Reviewer 3 requests additional analyses and consideration of whether the study adequately reflects current treatment recommendations.

Authors’ response) We sincerely thank you for your thoughtful and constructive comments. We have carefully addressed all concerns raised in the revised manuscript. Specifically, we have provided full information on the standardized prompts used for each LLM in S2 Table and clarified in the Methods section. The process of determining concordance between physician and LLM recommendations has been clearly described. To address potential bias associated with the use of overall survival as the outcome, we conducted additional analyses including a 1-year landmark analysis, stabilized inverse probability of treatment weighting (IPTW), and doubly robust (augmented IPTW) Cox models; these adjustments confirmed the robustness of our findings and minimized immortal-time and selection bias. In response to Reviewer 3’s comments, we performed additional analyses and sensitivity checks, including era-stratified analyses to account for secular changes in treatment practice, as well as a tier-based evaluation distinguishing guideline adherence from therapeutic escalation effects. Through these revisions, we have enhanced methodological transparency, clarified analytical procedures, mitigated potential biases, and contextualized our findings. We believe the revised manuscript fully addresses the reviewers’ and editor’s concerns and substantially strengthens the study.

After discussing the paper with the editorial team and an academic editor with relevant expertise, I'm pleased to invite you to revise the paper in response to the reviewers' comments. We plan to send the revised paper to some or all of the original reviewers, and we cannot provide any guarantees at this stage regarding publication.

Please further note that any claims of clinical utility of LLMs must be removed given that the study is not a prospective trial. The Conclusions section of the Abstract must be revised accordingly, as well as the framing of concordance of physician and LLM recommendations in the Abstract.

Authors’ response) We have carefully addressed the Editor’s request regarding the phrasing of clinical utility. Statements implying clinical utility of large language models have been removed or rephrased to emphasize associative findings rather than interventional implications (page 20, lines 443–445). The Conclusions section of the Abstract has been revised accordingly to reflect that the results demonstrate associations between LLM–physician concordance and survival outcomes rather than evidence of prospective clinical benefit (page 3, lines 78–80).

The technological limitations of the methods must be stated in the Abstract (Methods & Findings section), and the overall limitations must be included in the last section of the Author Summary.

Authors’ response) We have addressed the Editor’s request by adding explicit statements on both the technological and overall limitations. In the Abstract (Methods & Findings section), we added a sentence noting that the LLMs operated solely on structured clinical variables without access to imaging data, which may limit the precision of their treatment recommendations (page 3, lines 74–76). In the Author Summary, we also added a final sentence summarizing the overall study limitations (page 5, lines 114–115).

New code generated for this study must be deposited in a publicly accessible database. Please also refer to our Data Availability requirements and specify all restrictions to data access and the process for data access. Please note that 'upon reasonable request' is not an approved process for data access.

Authors’ response) We have revised the Data Availability statement in accordance with the journal’s policy. The phrase “upon reasonable request” has been removed (page 1, lines 20–25).

Please include a completed CHART and/or TRIPOD-LLM checklist.

Authors’ response) We have included a completed CHART checklist in the S1 Table, as requested.

In addition to these revisions, you may need to complete some formatting changes, which you will receive in a follow up email. A member of our team will be in touch with a set of requests shortly. If you do not receive a separate email within a few days, please assume that checks have been completed, and no additional changes are required.

Authors’ response) We have carefully reviewed and completed all the requested technical and formatting updates. The Data Availability Statement has been revised, and the funding sources and grant information have been verified for consistency. A statement regarding the funders’ role has also been included. All figure files have been provided in the required high-resolution .tif format. In addition, a full list of legends for the supporting information files has been added after the References section.

When you upload your revision, please include a point-by-point response that addresses all of the reviewer and editorial points, indicating the changes made in the manuscript and either an excerpt of the revised text or the location (eg: page and line number) where each change can be found. Please also be sure to check the general editorial comments at the end of this letter and include these in your point-by-point response. When you resubmit your paper, please include a clean version of the paper as the main article file and a version with changes tracked as a marked-up manuscript. It may also be helpful to check the guidelines for revised papers at http://journals.plos.org/plosmedicine/s/revising-your-manuscript for any that apply to your paper.

Authors’ response) We have carefully followed all instructions for the revised submission. A detailed point-by-point response addressing every reviewer and editorial comment has been included, indicating all corresponding changes in the manuscript along with page and line numbers. Both a clean version and a marked-up version with tracked changes have been uploaded as requested.

[Comments from the academic editor]

1. The inputs for the LLM models are unclear and likely insufficient to make accurate treatment recommendations (which drove some of the concordance). For example, eligibility for ablation and resection can be influenced by tumor location, which would be difficult for the models to take into account without being fed full images.

Authors’ response) We appreciate this insightful comment. We agree that the absence of imaging information (e.g., tumor location or vascular proximity) limits the precision of treatment recommendations, particularly for procedures such as resection or ablation. However, as this study utilized data from a nationwide registry (the Korean Primary Liver Cancer Registry), detailed radiologic parameters not consistently available. All three models were queried using standardized clinical inputs consistent with the AASLD/EASL decision variables, including tumor number and size, vascular invasion, extrahepatic spread, Child-Pugh class, and ECOG status. Thus, the models were intentionally restricted to information typically used in population-level decision algorithms rather than patient-specific imaging data. We have clarified this methodological constraint in the revised Methods section (page 8, lines 187–191) and have added a note in the Discussion section (page 21, lines 473–478) that future multimodal frameworks integrating imaging and text-based clinical data could enhance the contextual precision of LLM-driven treatment recommendations. In addition, as this study aimed to evaluate the real-world feasibility of LLM-based decision support across a nationwide registry, incorporating full imaging data for each patient would not only be practically infeasible but also ethically inappropriate due to privacy constraints. The study therefore focused on structured clinical variables that are routinely accessible in population-level datasets, aligning with the intended scope of assessing the contextual appropriateness of LLMs in typical clinical scenarios.

2. BCLC staging is heterogeneous and several studies have shown discordance between treatment practices and guideline recommendations (including impact on survival).

Authors’ response) We appreciate this important comment. We acknowledge that the BCLC staging system encompasses a heterogeneous population, and that real-world treatment decisions often diverge from guideline-recommended strategies due to individual variations in hepatic reserve, tumor biology, and institutional expertise. Indeed, several previous studies have reported that such deviations may influence survival outcomes across BCLC stages.

In our analysis, these heterogeneities are reflected in the stage-specific impact of LLM–physician concordance: concordant (guideline-aligned) treatments were associated with better survival in BCLC-A, whereas in BCLC-C, non-concordant treatments showed superior outcomes. We interpret this as evidence that real-world physicians frequently individualize treatment in advanced HCC—applying locoregional or combination therapies beyond strict guideline recommendations—whereas LLMs adhere rigidly to standardized protocols.

We have clarified this contextual interpretation in the revised Discussion section (page 20, lines 431–433), emphasizing that our findings highlight both the strength and limitation of guideline-based reasoning in heterogeneous clinical populations.

3. The cohort and guideline recommendations are now older (with both AASLD and EASL being updated in the past 1-2 years), and several treatment regimens now evolving over time including introduction of immunotherapy and increasing use of TARE.

Authors’ response) We appreciate this important comment. We fully acknowledge that treatment paradigms for HCC have rapidly evolved in recent years, including the introduction of immunotherapy combinations and broader adoption of TARE. However, our study intentionally aligned the LLMs with the AASLD and EASL guidelines that were current at the time of patient enrollment (2008–2020), as shown in Figure 1. This design ensured temporal consistency between the clinical data and the reference standards used for LLM training and evaluation. This approach allowed us to evaluate the intrinsic reasoning capacity of LLMs within guideline-defined contexts, independent of evolving treatment availability.

To further address potential secular effects, we performed additional era-stratified sensitivity analyses (2008–2012, 2013–2016, 2017–2020). As presented in S6 Table, hazard ratios for overall survival were consistent across calendar periods within each BCLC stage, indicating that temporal shifts in treatment availability or practice did not materially affect the observed associations between LLM–physician concordance and survival outcomes. These results and corresponding descriptions have been incorporated into the revised Methods and Results sections (page 13, lines 281–284).

We have clarified this rationale in the revised Methods section (page 8, lines 181–182) and have added a note in the Discussion (page 21, lines 478–481) emphasizing that future studies using updated cohorts and incorporating contemporary therapeutic options will be essential to assess the adaptability of LLM-based treatment reasoning in the modern immunotherapy era.

4. Higher proportion of HBV and Child Pugh A patients decreases generalizability to many Western centers.

Authors’ response) We agree that the predominance of hepatitis B–related and Child-Pugh A patients in our nationwide registry may limit direct generalizability to Western populations, where HCV, MASLD, and more advanced liver dysfunction are more prevalent. However, this cohort accurately represents the epidemiologic profile of HCC in East Asia, where HBV remains the major etiology. Although not shown in the revised manuscript, subgroup analyses restricted to non-HBV patients demonstrated similar stage-dependent trends in overall survival (particularly within BCLC stage A and C), supporting the robustness of our findings.

The goal of this study was to evaluate the conceptual validity of LLM-based clinical reasoning using a large, well-characterized dataset rather than to model a specific regional distribution. We have acknowledged this as a limitation in the revised Discussion section (page 21, lines 481–483).

5. Table 3 is informative but it would be better if there were details of why treatments differed. For example, why were 843 patients treated with TACE instead of resection? Without knowing if this was appropriate (e.g. portal hypertension or tumor location) or inappropriate (lack of seeing a surgeon), it's hard to understand the clinical relevance.

Authors’ response) We thank the reviewer for this valuable comment. We agree that understanding the clinical rationale behind treatment discordance—such as the presence of portal hypertension, tumor location, or patient comorbidities—would provide deeper clinical context. However, as the nationwide registry did not capture granular variables related to surgical feasibility or multidisciplinary decision processes, our analysis focused on quantifying patterns of divergence between LLM-generated and real-world treatment strategies rather than determining their appropriateness at an individual level. Importantly, evaluating the appropriateness of each individual treatment decision was beyond the scope of this registry-based validation study, which aimed to assess population-level patterns of concordance rather than case-level clinical judgment. We have clarified this scope in the revised Methods section (page 8, lines 187–191) and added a statement in the Discussion (page 21, lines 473–478) noting that future investigations integrating imaging and functional data will be required.

[Comments from the reviewers]

Reviewer #1: "Evaluating the Clinical Utility of Large Language Models for Hepatocellular Carcinoma Treatment Recommendations" explores the use of large language models (LLMs), for the management of hepatocellular carcinoma (HCC) treatment. This was done by comparing LLM-generated recommendations against actual clinical decisions and survival outcomes (overall survival; OS), on some 13000 patients from the Korean Primary Liver Cancer Registry. It was concluded that LLM recommendations had clinical utility in early-stage HCC, but not advanced-stage HCC. While the findings are informative towards the potential use of LLMs as an adjunctive tool towards HCC management, some initial concerns might be addressed:

Authors’ response) We sincerely thank the reviewer for the thoughtful and constructive evaluation of our manuscript. We are pleased that the reviewer recognized the potential clinical relevance of LLMs as adjunctive tools for HCC management. We have carefully addressed all the comments raised and revised the manuscript accordingly to improve its clarity, methodological transparency, and clinical interpretability.

1. In the Generation and Evaluation of LLM-Based Treatment Recommendations section, it is stated that patients were classified as "matched" if the LLM recommendation corresponded to the actual treatment received. It might be clarified as to whether an exact textual match was required for the recommendation, and if not, how non-exact matches were converted.

Authors’ response) We thank the reviewer for this helpful comment. The classification of “matched” cases was based on clinically equivalent treatment categories rather than exact textual agreement. LLM-generated recommendations were manually reviewed and mapped to the standardized treatment categories in the registry (e.g., surgical resection, local ablation, transarterial chemoembolization, systemic therapy, or best supportive care). We have clarified this definition in the revised Methods section (page 9, lines 195–196).

2. Related to the above, the standardized prompts used for the LLMs should be included in supplementary material, possibly with example inputs and generated outputs (and corresponding physician recommendations). In particular, did the LLM prompts constrain the possible recommendations?

Authors’ response) We thank the reviewer for this valuable suggestion. All three LLMs were queried using identical, guideline-based standardized prompts that described each patient’s clinical profile (demographics, tumor characteristics, liver function, and performance status) and explicitly instructed the model to recommend a treatment according to AASLD/EASL guidelines current at the time of diagnosis. Importantly, the prompts did not constrain or restrict the possible treatment options—the models generated responses freely within their internal reasoning, without predefined choices or forced outputs. To improve transparency and reproducibility, we have added representative examples of the standardized prompts, model-generated outputs, and corresponding physician treatments to the S2 Table and clarified this description in the revised Methods section (page 9, lines 198–199).

3. In the Impact of concordance between LLM-physician on OS section, it is stated that in general, patients whose treatments matched LLM recommendations (i.e. the LLM assessment and original clinical assessment are in agreement) exhibited significantly better survival outcomes, particularly for early-stage BCLC-A and BCLC-B patients. However, for advanced-stage BCLC-C patients, matched cases had significantly worse OS (HR=2.27) compared to mismatched cases.

Firstly, the claim of clinical utility for LLM in early-stage cases but not advanced-stage cases should be further justified. In particular, the counterfactual outcome where the LLM recommendation is applied (instead of the original recommendation in real life) remains unknown; considering this, assertions about the true utility of the LLM recommendations in mismatched cases (relating to claims that "...they lack contextual awareness for complex clinical situations requiring individualized care") may not be warranted. This issue should be carefully considered if deemed appropriate.

Authors’ response) We appreciate this important comment and fully agree that the counterfactual outcome—had the LLM-generated recommendations been applied in real-world practice—cannot be determined in this retrospective design. Accordingly, our findings should be interpreted as associations between LLM–physician concordance and survival outcomes, rather than as evidence of causal or interventional utility.

Our primary goal was to explore whether alignment between guideline-based LLM reasoning and real-world clinical decisions correlates with prognosis, not to evaluate the direct effectiveness of LLM-generated recommendations. The observation that matched cases showed better outcomes in early-stage HCC but worse outcomes in advanced-stage disease likely reflects the complexity of real-world clinical reasoning rather than the intrinsic performance of the LLMs.

We have revised the Discussion (page 20, lines 443–445) to clarify that the study demonstrates an association, not evidence of clinical efficacy, and that the remarks on contextual awareness in advanced-stage disease are exploratory and intended for conceptual understanding. We have also modified the Abstract conclusion (page 3, lines 80–82) to soften the language accordingly, reflecting that the observed patterns may indicate limited contextual awareness rather than a definitive lack of such awareness.

4. Related to the above, the observation that LLM and physician concordance for advanced-stage patients was associated with worse OS might be analyzed further. The following sections (and Table 3) attempt to explain causes and patterns of discordance between LLMs and physicians, but do not appear to discuss and analyze patterns of concordance, especially for BCLC-C cases. This is important since the presumed use of LLMs as an adjunctive tool would appear to involve identifying cases for deeper assessment by physicians.

Authors’ response) As suggested, we have analyzed the concordant BCLC-C subgroup using data summarized in S10 Table. Exploratory profiling revealed that concordant patients generally exhibited poorer hepatic reserve—including higher bilirubin, lower albumin, and more frequent ECOG ≥ 2 or ascites—indicating that LLM–physician concordance primarily reflected adherence to guideline-directed therapy among clinically fragile patients with limited hepatic function. This selection bias likely contributed to the observed poorer survival in concordant BCLC-C cases.

Importantly, this pattern does not suggest that LLM recommendations were harmful but rather highlights the contextual complexity of advanced-stage HCC, where guideline logic often applies to patients already predisposed to poor outcomes. We have also noted in the Discussion (page 20, lines 425–429) that such concordant yet high-risk profiles may, in future, serve as LLM-assisted prompts for multidisciplinary reassessment, helping identify patients who might benefit from individualized evaluation rather than automatic application of guideline-based systemic therapy.

5. The use of overall survival (OS) as a metric for quantifying the clinical relevance of LLM recommendations may have caveats. In particular, it does not appear to take into account the actual expected survival outcome of the patient, given their specific circumstances (e.g. liver function, tumor factors, etc.). As such, if an LLM (or any other statistical or machine learning model) biases its recommendations towards patient profiles with higher survival rates, then its OS metric for matched cases would automatically improve as a result.

As a simple concrete example, consider a HCC population that has 50% of cases with surgical resection as best treatment (with a 90% survival rate), and the other 50% of cases with best supportive case as best treatment (with a 10% survival rate). Then, an LLM that simply always recommends surgical resection would achieve a very high OS for matched cases, whether or not the recommendation is logically justified (which is often not true, since this LLM always gives the same recommendation). However, this LLM would be concluded to have high clinical utility from the OS outcome.

If this understanding is correct, authors might consider also incorporating the actual expected survival outcome of patients, in analyzing the clinical utility of the LLMs.

Authors’ response) We appreciate the reviewer’s thoughtful concern that using overall survival (OS) for “LLM-matched” cases could be confounded if a model tends to recommend treatments for patients with intrinsically better prognosis. We agree that naive comparisons of OS can be misleading. In our study, however, we explicitly mitigated this issue in three ways:

1) Risk adjustment and stratification.

All survival analyses were performed with BCLC-stage stratification and multivariable Cox models including key liver function and tumor covariates. In fully adjusted, stage-specific models, concordance was beneficial only in BCLC-A (e.g., ChatGPT HR 0.743, 95% CI 0.665–0.831) but harmful in BCLC-C (HR 1.650, 95% CI 1.523–1.787). If the observed effects were driven purely by a bias toward patients with better baseline prognosis, we would expect uniformly favorable OS in matched cases across all stages, which was not observed.

2) Clinical mechanism is consistent with covariate patterns.

Decision-tree and feature-importance analyses show that physicians prioritized liver function (e.g., platelet count, INR) whereas LLMs emphasized tumor burden (size, portal vein invasion, multiplicity). In BCLC-A, mismatched cases had poorer hepatic reserve (lower albumin, lower platelets, higher INR), suggesting that clinicians appropriately avoided curative options that LLMs—being guideline-driven—often proposed. In contrast, in BCLC-C, mismatched cases had better liver function and less metastatic burden than matched cases, consistent with clinicians favoring loco-regional control in selected patients despite guideline-suggested systemic or palliative options. These patterns argue against a simple “LLM selects healthier patients” explanation.

3) Within-treatment sensitivity analysis.

To address the reviewer’s suggestion directly, we added S16 Table, reporting median OS within each actual treatment and the corresponding subset concordant with each LLM. If LLMs were merely steering toward inherently long-survival treatments, median OS for “concordant” cases should systematically exceed the treatment-level baseline. Instead, results were mixed—e.g., in BCLC-A, surgical resection medians were similar between all patients and LLM-concordant groups, while RFA medians were lower for ChatGPT but higher for Gemini. In BCLC-C, best-supportive-care medians were similar or even slightly worse when concordant. These findings argue against uniform survival inflation in matched cases. This additional analysis has been incorporated into the Results section (page 16, lines 358–363) to clarify the robustness of the survival findings.

Taken together, our conclusions do not rest on unadjusted OS; they are consistent across risk-adjusted, stage-stratified, and within-treatment analyses. The stage-specific pattern observed—favorable in early-stage and divergent in advanced-stage HCC—supports that the findings reflect true clinical context and differences in decision logic rather than selection bias. Future analyses incorporating individualized survival prediction models could further disentangle whether LLM–physician concordance reflects baseline prognostic alignment or true clinical reasoning consistency.

6. Related to the above, complete matrices showing the prevalence and OS of all possible recommendation combinations (similar to Figure 4c) might be included as a figure. The OS for each actual treatment might also be included as a table.

Authors’ response) We appreciate the reviewer’s constructive suggestion. In response, we newly added S13 Fig, which presents complete prevalence matrices of all possible treatment recommendation combinations across the three LLMs (page 15, lines 347–348).

However, an additional OS matrix including survival outcomes for each combination was deemed potentially confusing for readers due to its multidimensional structure and overlapping strata. Moreover, several matrix cells contained only a very small number of patients, which could further complicate interpretation and lead to unstable survival estimates.

Instead, as described above, we incorporated a complementary within-treatment survival analysis in S16 Table (page 16, lines 358–361), reporting median OS for each actual treatment and the corresponding LLM-concordant subset. This approach provides a clearer and more interpretable assessment of survival patterns without overcomplicating data presentation.

7. It might be clarified as to whether physician treatment recommendations are always followed, or whether other considerations (e.g. cost, patient choice) may result in physician recommendation and actual treatment being discordant. If the latter, the prevalence of such discordant treatments might be commented on.

Authors’ response) We appreciate the reviewer’s insightful comment. In the Korean Primary Liver Cancer Registry, treatment information reflects the actual therapy administered, as abstracted from hospital medical records after completion of therapy. The registry does not separately record physician recommendations that were not ultimately carried out. Therefore, all treatment data used in our analysis correspond to therapies that were actually performed, rather than proposed or planned. We have clarified in the Methods section (page 8, lines 173–175) that the treatment data reflect completed therapies administered in this cohort, rather than physician-intended recommendations.

8. In the Factors underlying LLM-physician discordance section, it is stated that decision tree models were constructed to assess key clinical variables influencing treatment selection in both physician decisions and LLM-generated recommendations. However, Figure 3 appears to show feature importances of various factors. The construction methodology of the decision trees for both LLM and physician recommendations should thus be described in detail, possibly in supplementary material. If possible, example decision trees and their corresponding recommendation should also be provided.

Authors’ response) We have now provided a detailed description of the decision tree construction process in the Methods section (page 10, lines 226–228). Specifically, decision tree classifiers were trained separately for clinician decisions and each LLM recommendation using identical clinical covariates and Gini impurity–based recursive partitioning. The trees were pruned using cross-validation to enhance interpretability and avoid overfitting. In addition, a simplified decision tree model has been newly analyzed and included as S7 Fig in Results section (page 14, lines 315–316), illustrating representative decision pathways for clinicians and each LLM.

Reviewer #2: The authors task three generative AI chatbots with providing treatment advice for hepatocellular carcinoma patients, comparing this output to actual decisions made by the patients' physicians. This is an interesting application and of interest to clinicians and researchers wondering about the potential applications of AI. Reporting could be strengthened using CHART (see point 1), and more detail is necessary in the Methods section. Other than this, my comments are mostly minor.

1. The CHART tool (https://www.equator-network.org/reporting-guidelines/reporting-guideline-for-chatbot-health-advice-studies-the-chart-statement/) has been designed for studies of generative AI providing advice to clinicians or patients. The manuscript would be strengthened by adhering to this. Guidance is provided in any of six peer reviewed articles, and a website is available to automatically generate a checklist.

Authors’ response) We thank the reviewer for this helpful recommendation. We have reviewed the CHART (Chatbot Health Advice Reporting Template) guideline and confirmed that our study adheres to its major domains, including model transparency, description of prompts, clinical task definition, and evaluation of recommendation accuracy and clinical relevance. To ensure comprehensive and transparent reporting, we have completed the CHART checklist and included it as S1 Table (page 8, lines 175).

2. In the Methods, the models should be explicitly described as proprietary and 'out-of-the-box'/unmodified (presuming the authors made no fine-tuning or other modification beyond the information in the prompts described).

Authors’ response) We thank the reviewer for this important clarification. All LLMs used in this study (ChatGPT 4o, Gemini 2.0, and Claude 3.5) were proprietary, closed-source models accessed via their respective public interfaces. No fine-tuning, parameter modification, or model customization was performed beyond the standardized prompts described in the Methods. We have revised the Methods section to explicitly describe the models as proprietary and unmodified (page 8, lines 182–184).

3. Was any formal prompt engineering process undertaken, or how were prompts designed? Who designed them, were multiple prompts tested, and how was a final prompt selected? The AASLD and EASL guidelines should also be cited.

Authors’ response) We appreciate the reviewer’s insightful comment. A formal iterative prompt engineering process was not conducted; instead, standardized prompts were systematically designed by the study investigators (hepatology specialists) to ensure reproducibility and alignment with clinical reasoning steps. Each component of the final prompt set was systematically described in S2 Table in the revised manuscript. Prompts were reviewed and finalized through internal consensus among investigators before model inference, and all LLMs were queried using identical instructions. The prompts were based on and consistent with the latest AASLD (2018) and EASL (2018, 2022) HCC management guidelines, which are now explicitly cited in the Methods section (page 8, lines 182).

4. More information regarding evaluation is necessary in the Methods, perhaps in an additional subsection. Who decided whether treatment was concordant with actual treatment; did more than one judge conduct assessment; and how were any discrepencies resolved? Did treatment have to match exactly, or was the same agent / type of regime / duration of treatment sufficient? In short, precisely what counted as matching or mismatching, and why?

Authors’ response) We thank the reviewer for this valuable suggestion. We have now clarified in the Methods section how concordance between LLM-generated and actual treatments was assessed. Specifically, two hepatologists independently reviewed all treatment labels to determine whether the LLM-recommended strategy matched the real-world treatment. Matching was defined at the level of clinically equivalent treatment categories (e.g., resection, local ablation, TACE, systemic therapy, or best supportive care), rather than requiring an exact textual or regimen-level identity. Minor variations in agent type, dosage, or duration were not classified as mismatches, since these details were not consistently captured in the registry dataset. This clarification has been added to the Methods section (page 9, lines 193–199) in the revised manuscript.

5. The process of modelling overall survival following LLM recommendations is not clear--and currently reads almost as though patients underwent treatment based on the LLM suggestion (which is not the case). It must be crystal clear that survival analyses stratified patients based on concordance between LLM and physician decisions.

Authors’ response) We thank the reviewer for this important clarification. We fully agree that no patient in this study received treatment based on LLM-generated recommendations. As described in the revised Methods section, all treatments were administered according to real-world physician decisions recorded in the national registry. The survival analyses were conducted retrospectively by stratifying patients according to the concordance or discordance between these physician-administered treatments and LLM-simulated recommendations. This approach allowed us to evaluate whether alignment with LLM-generated strategies—had they been applied—was associated with different survival outcomes, rather than implying any real clinical intervention based on LLM output. We have revised the Methods section (page 9, lines 204–208) to clarify this distinction.

6. In the Results, references to 'curative therapy' should read 'potentially curative therapy'.

Authors’ response) We thank the reviewer for this helpful comment. We agree that the term “potentially curative therapy” is more appropriate. All references to “curative therapy” in the Results section have been revised accordingly (page 9, lines 325, 344, and 349).

7. In the Results subsection "Factors underlying LLM-physician discordance' there is too much speculation, generally at the end of each paragraph. These speculations as to why discrepancies are observed should be explained in the Discussion as there are not proven or disproven by the study.

Authors’ response) We thank the reviewer for this helpful suggestion. We agree that some sentences in the Results subsection “Factors underlying LLM–physician discordance” were speculative. Since these interpretations are already sufficiently addressed in the Discussion, we have removed the corresponding speculative statements from the Results section to maintain a purely descriptive presentation.

8. The Discussion does not explore alternative hypotheses for why discrepancies between physicians and generative AI is observed. For instance, patients may choose not to or to have surgery if offered, which would be expected to lead to discrepancy with guideline recommendations.

Authors’ response) We thank the reviewer for this valuable comment. We agree that several alternative explanations could underlie the discrepancies observed between physicians and LLMs. Beyond differences in clinical priorities, these inconsistencies may also arise from patient-level factors such as treatment preference, surgical eligibility, or comorbid conditions; from limitations in registry data, including the absence of imaging or psychosocial information; and from model-related issues, where LLMs depend primarily on textual guideline patterns without sufficient contextual reasoning. We have expanded the Discussion section (page 20, lines 446–455) to incorporate these possible alternative hypotheses.

9. The limitations paragraph is far too brief. The observational design precludes any causal inference or conclusions regarding the utility of generative AI due to selection bias as well as apple to oranges comparisons (actual versus modelled survival) and neglect of patient opinions/decisions regarding treatment. A call for prospective studies of AI-enhanced decision-making versus conventional care directed by the multidisciplinary team would be appropriate.

Authors’ response) We thank the reviewer for this insightful comment. We acknowledge that the observational design limits any causal interpretation of the association between LLM-generated recommendations and survival outcomes. We have expanded the Limitations paragraph (page 21, lines 473–486) to acknowledge the potential for selection bias, the non-comparability of modeled versus actual survival outcomes, and the absence of patient decision factors. In addition, we have added a statement emphasizing the need for prospective studies comparing AI-assisted decision-making with conventional multidisciplinary care.

Reviewer #3: This large registry study (2008-2020) compares LLM‑generated, guideline‑based treatment suggestions for HCC with real‑world management and outcomes. While stage‑stratified results are intriguing, the current design cannot disentangle adherence effects from baseline differences and secular changes in practice.

- Major comments -

1. Confounding by indication & time‑related bias (primary concern).

The survival gap between "match" and "mismatch" groups likely reflects baseline imbalances and feasibility rather than the act of following an LLM recommendation. Please (a) define a clear time‑zero and a prespecified treatment window (e.g., 60-90 days) and use either a landmark analysis (follow‑up starts at the landmark) or a time‑dependent treatment model to avoid immortal‑time bias; (b) reframe analyses within each BCLC stage and recommended therapy as the effect of adhering to that therapy; (c) estimate stabilized IPTW (or overlap weights) for adherence using only pre‑treatment covariates (age, ECOG, Child‑Pugh/ALBI, portal‑hypertension surrogates, tumor burden, AFP, calendar year, and—if available—center). Show balance diagnostics (SMD <0.10) and fit a weighted Cox (Aalen-Johansen if competing risks are relevant). Provide sensitivity analyses with doubly‑robust estimators (AIPW/TMLE). Temper causal language accordingly.

Authors’ response) We appreciate the reviewer’s insightful comment. In the present analysis, time zero was defined as the date of treatment initiation. Because treatment initiation varied across patients, this design could theoretically introduce immortal-time bias if patients who survived longer were more likely to receive a particular treatment and thus be classified as “matched.”

To address potential immortal-time bias, we additionally conducted a 1-year landmark analysis, restricting the cohort to patients who survived at least 12 months after diagnosis and initiating follow-up from that landmark. As shown in the revised S3–5 Fig, the stage-dependent pattern of survival differences between LLM-matched and mismatched cases remained consistent even after landmark adjustment, indicating that the observed associations were not solely driven by differences in treatment timing or feasibility.

In addition, within each BCLC stage, we applied stabilized inverse probability of treatment weighting (IPTW) to minimize confounding factors. After weighting, covariate balance was well achieved across all models (absolute SMD < 0.10 for all covariates; S6 Fig), and results from both IPTW and doubly robust (augmented IPTW) Cox models (S7–8 Table) confirmed that the survival associations persisted across alternative causal specifications. Collectively, these analyses support that the observed survival differences represent robust, stage-specific associations rather than artifacts of immortal-time bias or baseline confounding.

These additional analyses have been newly described in the Methods (page 9, lines 212–213) and Results sections (page 14, lines 290–306) of the revised manuscript.

2. Clinical perspective: guidelines vs individualised care, and therapeutic hierarchy.

Non‑adherence to AASLD/BCLC is not inherently detrimental; in complex, multiparametric HCC, individualised decisions outside rigid algorithms can yield better outcomes (e.g., Lancet Oncol 2023; doi: 10.1016/S1470-2045(23)00186-9). Your own findings suggest that the apparent advantage of certain LLM cohorts stems less from "following guidelines" and more from recommending higher‑tier, curative‑intent options (resection, transplant, ablation) relative to real‑world choices.

Actionable analyses:

Classify treatments into ordered tiers (curative‑intent: resection/transplant/ablation; non‑curative: TACE; systemic; best supportive care).

Decompose effects into (i) adherence to the specific recommendation and (ii) tier escalation (LLM‑recommended therapy higher vs equal/lower tier than real‑world).

Within‑stage, estimate the effect of tier escalation with IPTW and report both survival and treatment‑receipt outcomes (e.g., odds of receiving curative‑intent therapy). This will clarify whether LLMs are useful because they privilege higher‑value therapies, not simply because they mirror guidelines.

Authors’ response) We thank the reviewer for this insightful comment. To address this, we performed an additional tier-based decomposition analysis that classified all treatments into four ordered categories — (1) curative-intent (resection, transplantation, ablation), (2) locoregional non-curative (TACE, radioembolization), (3) systemic therapy, and (4) best supportive care — and evaluated within-stage, IPTW-adjusted effects of tier escalation on overall survival across LLMs.

First, stage-specific transition patterns revealed distinct tendencies across disease stages (S17 Table). In early-stage (BCLC A) disease, LLMs more frequently recommended higher-tier, curative-intent therapies such as resection or ablation compared with real-world practice. Conversely, in advanced-stage (BCLC C) disease, LLMs tended to recommend lower-tier or less-aggressive options more often than clinicians, reflecting a more conservative stance when treatment feasibility was low.

Next, within each BCLC stage, tier escalation (LLM recommending a higher-tier therapy relative to actual treatment) was associated with significantly improved overall survival after IPTW adjustment (S18 Table). Together, these findings clarify that the survival advantage observed in certain LLM cohorts reflects their ability to prioritize clinically appropriate, higher-value or curative-intent therapies when feasible, rather than mere adherence to guideline concordance. We additionally incorporated this supplementary analysis into the Results section (page 16, lines 361–366).

3. Secular trends and center effects.

Given major shifts in locoregional techniques and systemic therapy across 2008-2020, adjust for calendar time (splines or eras) and, if available, center; add era‑stratified sensitivity analyses, especially in BCLC‑C.

Authors’ response) We appreciate the reviewer’s suggestion regarding potential secular and center effects. To account for temporal changes in treatment paradigms, each model was provided with identical clinical input variables alongside standardized prompts explicitly referencing the AASLD and EASL guidelines that were current at the time of each patient’s diagnosis. Center-specific analyses were not feasible due to registry anonymization; however, we additionally conducted era-stratified sensitivity analyses (2008–2012, 2013–2016, 2017–2020) to assess the robustness of model performance and survival outcomes across calendar periods. As shown in S6 Table, the direction and magnitude of hazard ratios for each BCLC stage were largely consistent across eras, indicating that the associations between LLM–physician concordance and overall survival remained stable over time despite secular changes in treatment practice. These results and corresponding descriptions have been added to the Results section of the revised manuscript (page 13, lines 281–285).

4. Minimal transparency/reproducibility fixes.

Align the Data & Code Availability statement with PLOS policy; deposit de‑identified analysis‑ready data (or an auditable access pathway) and all scripts/prompts/parameters. Document LLM versioning (model versions, inference dates, decoding parameters, number of runs/seeds) and provide raw outputs to ensure reproducibility.

Authors’ response) We thank the reviewer for emphasizing the importance of transparency and reproducibility. The Data Availability statement was already included in the original manuscript. Specifically, all standardized prompts and inference parameters have been added to S2 Table, and additional methodological details regarding LLM inference have been described in the Methods section (page 9, lines 195–199). Furthermore, the raw outputs generated by the three LLMs (ChatGPT-4o, Gemini 2.0, and Claude 3.5) will be provided as de-identified .xlsx file (S1 Data) upon acceptance to facilitate independent verification and reproducibility (page 9, lines 205).

- Minor comments -

1) Reconcile any table-figure HR inconsistencies in BCLC‑B; improve figure resolution and provide vector graphics with numbers‑at‑risk.

Authors’ response) We appreciate the reviewer’s comment. All potential inconsistencies between tables and figures for the BCLC-B subgroup have been carefully reviewed. Figure resolution has been improved, and numbers-at-risk have been added to all Kaplan–Meier plots in the revised manuscript.

2) Standardise TACE terminology across text and figures.

Authors’ response) We thank the reviewer for the helpful suggestion. The terminology for TACE has been reviewed and standardized across the text, tables, and figures in the revised manuscript.

Any attachments provided with reviews can be seen via the following link:

--------------------------------------------------------- ---

General editorial requests:

(Note: not all will apply to your paper, but please check each item carefully)

* We ask every co-author listed on the manuscript to fill in a contributing author statement, making sure to declare all competing interests. If any of the co-authors have not filled in the statement, we will remind them to do so when the paper is revised. If all statements are not completed in a timely fashion this could hold up the re-review process. If new competing interests are declared later in the revision process, this may also hold up the submission. Should there be a problem getting one of your co-authors to fill in a statement we will be in contact. Please do not add or remove authors without first discussing this with the handling editor. You can see our competing interests policy here: <http://journals.plos.org/plosmedicine/s/competing-interests>.

We will confirm that all co-authors have completed the contributing author statement and declared any potential competing interests in accordance with the journal’s policy.

* Please upload any figures associated with your paper as individual TIF or EPS files with 300dpi resolution at resubmission; please read our figure guidelines for more information on our requirements: http://journals.plos.org/plosmedicine/s/figures. While revising your submission, we strongly recommend that you use PLOS's NAAS tool (https://ngplosjournals.pagemajik.ai/artanalysis) to test your figure files. NAAS can convert your figure files to the TIFF file type and meet basic requirements (such as print size, resolution), or provide you with a report on issues that do not meet our requirements and that NAAS cannot fix.

After uploading your figures to PLOS's NAAS tool - https://ngplosjournals.pagemajik.ai/artanalysis, NAAS will process the files provided and display the results in the "Uploaded Files" section of the page as the processing is complete.

If the uploaded figures meet our requirements (or NAAS is able to fix the files to meet our requirements), the figure will be marked as "fixed" above. If NAAS is unable to fix the files, a red "failed" label will appear above.

When NAAS has confirmed that the figure files meet our requirements, please download the file via the download option, and include these NAAS processed figure files when submitting your revised manuscript.

We have reviewed the figure requirements and confirmed that all figures have been uploaded as individual high-resolution TIF files in accordance with the journal’s guidelines. We have processed all figure files using the PLOS NAAS tool as recommended. The figures were successfully converted and verified to meet the journal’s requirements for file type, resolution, and print size. The NAAS-processed figure files have been downloaded and included in the revised submission.

* Please ensure that the paper adheres to the PLOS Data Availability Policy (see http://journals.plos.org/plosmedicine/s/data-availability), which requires that all data underlying the study's findings be provided in a repository or as Supporting Information. For data residing with a third party, authors are required to provide instructions with contact information (web or email address) for obtaining the data. Please note that a study author cannot be the contact person for the data. PLOS journals do not allow statements supported by "data not shown" or "unpublished results." For such statements, authors must provide supporting data or cite public sources that include it.

We have revised the Data Availability statement to fully comply with the PLOS Data Availability Policy.

* We expect all researchers with submissions to PLOS in which author-generated code underpins the findings in the manuscript to make all author-generated code available without restrictions upon publication of the work. In cases where code is central to the manuscript, we may require the code to be made available as a condition of publication. Authors are responsible for ensuring that the code is reusable and well documented. Please make any custom code available, either as part of your data deposition or as a supplementary file. Please add a sentence to your data availability statement regarding any code used in the study, e.g. "The code used in the analysis is available from Github [URL] and archived in Zenodo [DOI link]" Please review our guidelines at https://journals.plos.org/plosmedicine/s/materials-software-and-code-sharing and ensure that your code is shared in a way that follows best practice and facilitates reproducibility and reuse. Because Github depositions can be readily changed or deleted, we encourage you to make a permanent DOI'd copy (e.g. in Zenodo) and provide the URL.

We have reviewed the journal’s policy regarding code and materials sharing. In accordance with the policy, all standardized prompts used for LLM inference and raw model outputs have been provided in the Supporting Information to ensure transparency and reproducibility.

* [EDITOR: CHECK FINANCIAL DISCLOSURES, COI, DAS, AND ETHICS STATEMENTS AND INCLUDE ANY NECESSARY REQUESTS]

* Please ensure that the study is reported according to the [XXXX] guideline and include the completed [XXXX] checklist as Supporting Information. When completing the checklist, please use section and paragraph numbers, rather than page numbers. Please add the following statement, or similar, to the Methods: "This study is reported as per [XXXX] guideline (S1 Checklist)."

We have included the completed CHART checklist as Supporting Information and added the following statement to the Methods section as requested.

FORMATTING - GENERAL

* Abstract: Please structure your abstract using the PLOS Medicine headings (Background, Methods and Findings, Conclusions). Please combine the Methods and Findings sections into one section.

We have reviewed the journal’s formatting requirements and confirmed that the Abstract has been restructured according to the PLOS Medicine format, using the Background, Methods and Findings, and Conclusions headings.

* At this stage, we ask that you include a short, non-technical Author Summary of your research to make findings accessible to a wide audience that includes both scientists and non-scientists. The Author Summary should immediately follow the Abstract in your revised manuscript. This text is subject to editorial change and should be distinct from the scientific abstract. Ideally each sub-heading should contain 2-3 single sentence, concise bullet points containing the most salient points from your study. In the final bullet point of 'What Do These Findings Mean?', please include the main limitations of the study in non-technical language. Please see our author guidelines for more information: <https://journals.plos.org/plosmedicine/s/revising-your-manuscript#loc-author-summary>.

We have added a short, non-technical Author Summary following the Abstract, in accordance with the journal’s guidelines.

* Please express the main results with 95% CIs as well as p values. When reporting p values please report as p<0.001 and where higher as the exact p value p=0.002, for example. Throughout, suggest reporting statistical information as follows to improve clarity for the reader "22% (95% CI [13%,28%]; p</=)". Please be sure to define all numerical values at first use.

We have reviewed the journal’s statistical reporting requirements and revised the manuscript accordingly.

* Please include page numbers and line numbers in the manuscript file. Use continuous line numbers (do not restart the numbering on each page).

We have added page numbers and continuous line numbers throughout the manuscript, as requested. The line numbering does not restart on each page.

* Please cite the reference numbers in square brackets. Citations should precede punctuation.

We have reviewed the reference formatting and revised all in-text citations to appear in square brackets, ensuring that citations precede punctuation in accordance with the journal’s style requirements.

FIGURES AND TABLES

* Please provide titles and legends for all figures and tables (including those in Supporting Information files).

We have reviewed all figures and tables, including those in the Supporting Information.

* Please define all abbreviations used in each figure/table (including those in Supporting Information files).

All abbreviations are clearly defined within each figure and table legend, as requested.

SUPPLEMENTARY MATERIAL

* Please note that supplementary material will be posted as supplied by the authors. Therefore, please amend it according to the relevant comments outlined here.

We have reviewed and amended the supplementary materials.

* Please cite your Supporting Information as outlined here: https://journals.plos.org/plosmedicine/s/supporting-information

We have reviewed the Supporting Information citation guidelines.

REFERENCES

* PLOS uses the numbered citation (citation-sequence) method and first six authors, et al.

We have reviewed and confirmed that all references follow the PLOS citation-sequence style, listing the first six authors followed by et al. as required.

* Please ensure that journal name abbreviations match those found in the National Center for Biotechnology Information (NCBI) databases (http://www.ncbi.nlm.nih.gov/nlmcatalog/journals), and are appropriately formatted and capitalised.

All journal name abbreviations have been checked with appropriate capitalization.

* Where website addresses are cited, please include the complete URL and specify the date of access (e.g. [accessed: 12/06/2023]).

No website addresses were cited in the manuscript.

* Please also see https://journals.plos.org/plosmedicine/s/submission-guidelines#loc-references for further details on reference formatting.

All references have been formatted in accordance with the PLOS Medicine reference guidelines.

[STUDY TYPE-SPECIFIC REQUESTS - DELETE SECTIONS AS NECESSARY]

OBSERVATIONAL STUDIES

* Abstract: Please include the study design, population and setting, number of participants, years during which the study took place (enrollment and follow up), length of follow up, and main outcome measures.

The Abstract has been reviewed accordingly. Given the nature of this study, we have followed the reviewer’s suggestion and added the completed CHART checklist as Supporting Information to enhance transparency and reporting quality.

* For all observational studies, in the manuscript text, please indicate: (1) the specific hypotheses you intended to test, (2) the analytical methods by which you planned to test them, (3) the analyses you actually performed, and (4) when reported analyses differ from those that were planned, transparent explanations for differences that affect the reliability of the study's results. If a reported analysis was performed based on an interesting but unanticipated pattern in the data, please be clear that the analysis was data driven.

We have reviewed the manuscript and confirmed that all required information regarding the study hypotheses, planned analytical methods, performed analyses, and explanations for any deviations has been clearly stated.
