# Supplementary material for: Evaluating the clinical utility of large language models for hepatocellular carcinoma treatment recommendations: A nationwide retrospective registry study
Source: PLoS Med. 2026 Jan 13;23(1):e1004855. doi: 10.1371/journal.pmed.1004855 (PMC12799000; doi:10.1371/journal.pmed.1004855)
Supplement: Attachment — Submitted filename: Response letter_2nd revision.docx [file pmed.1004855.s038.docx]

[Comments from the senior editor]

Thank you very much for re-submitting your manuscript "Evaluating the Clinical Utility of Large Language Models for Hepatocellular Carcinoma Treatment Recommendations" (PMEDICINE-D-25-03475R2) for review by PLOS Medicine. I have discussed the paper with my colleagues and the academic editor and it was also seen again by 3 reviewers. I am pleased to say that provided the remaining editorial and production issues are dealt with we are planning to accept the paper for publication in the journal. We ask you to address the remaining concerns of reviewers 1 and 2 in a revised manuscript, and please include a point-by-point response to the reviewers' comments and the editorial requests at the end of the email.

Authors’ response) Thank you very much for your positive evaluation of our revised manuscript and for the opportunity to further improve it. We sincerely appreciate the constructive feedback provided by the editors and the reviewers throughout the process. We have carefully reviewed the remaining comments from Reviewers 1 and 2 as well as the editorial requests. In the newly revised version, we have addressed all points in detail and incorporated the requested clarifications. A comprehensive point-by-point response to each comment is included below this letter. We are grateful for your guidance and are pleased to submit this revised manuscript for your consideration.

[Comments from the editors]

General:

*The Author Summary repeats phrasing in the Abstract. Please revise the Author summary and make accessible to the general reader.

We have revised the Author Summary and rewritten it in clearer, more accessible language for a general reader.

* Please confirm that your title complies with PLOS Medicine's style. Your title must be nondeclarative and not a question. It should begin with main concept if possible. "Effect of" should be used only if causality can be inferred, i.e., for an RCT. Please place the study design ("A randomized controlled trial," "A retrospective study," "A modelling study," etc.) in the subtitle (ie, after a colon).

We have revised the manuscript title to comply with PLOS Medicine’s style requirements. The title is now non-declarative and begins with the main concept. In addition, the study design has been placed in the subtitle, as recommended.

* Please confirm that your abstract complies with our requirements, including format (three sections: Background, Methods and Findings, and Conclusions) and providing all the information relevant to this study type https://journals.plos.org/plosmedicine/s/submission-guidelines#loc-abstract.

We confirm that the Abstract now complies with all PLOS Medicine requirements. It follows the prescribed three-section structure (Background, Methods and Findings, and Conclusions) and includes all essential elements for an observational registry study as outlined in the journal’s submission guidelines.

* Please ensure that the Introduction ends with a clear description of the study question or hypothesis.

We have confirmed that the Introduction ends with a clear statement of the study question and hypothesis, as recommended.

* Please ensure that all abbreviations are defined at first use throughout the text.

We confirm that all abbreviations are defined at first use.

* Please confirm that all numbers presented in the abstract are present and identical to numbers presented in the main manuscript text.

We have reviewed the manuscript and confirmed that all numbers reported in the Abstract are present in the main text and are identical to those in the manuscript.

* Please include the URLs for the funders in the funding statement.

We have updated the Funding statement to include the URLs for all funders, in accordance with PLOS Medicine’s requirements.

* Statistical reporting:

- Please report statistical information as follows to improve clarity for the reader ""22% (95% CI [13,28]; p</=)"".

We have updated the manuscript so that all confidence intervals follow the required bracket format.

- Please separate upper and lower bounds with commas instead of hyphens as the latter can be confused with reporting of negative values.

We have reviewed the manuscript and confirm that the requested formatting has been applied.

- Please repeat statistical definitions (HR, CI etc.) for each set of parentheses.

We have ensured that statistical terms (e.g., HR, CI) are explicitly defined across all tables.

* In the abstract, please include the important dependent variables that are adjusted for in the analyses.

We have reviewed the Methods and findings section of the Abstract.

"* PLOS defines the “minimal data set” to consist of the data set used to reach the conclusions drawn in the manuscript with related metadata and methods, and any additional data required to replicate the reported study findings in their entirety. Authors do not need to submit their entire data set, or the raw data collected during an investigation. Please submit the following data:

The values behind the means, standard deviations and other measures reported;

The values used to build graphs;

The points extracted from images for analysis.

We have selected and submitted the required data in accordance with the journal’s guidelines.

* The Data Availability Statement (DAS) in the manuscript metadata requires revision as it does not match the restricted data availability stated in the manuscript. If the data are not freely available, please describe briefly the ethical, legal, or contractual restriction that prevents you from sharing it. Please also include an appropriate contact (email address) for inquiries (this cannot be a study author).

We have reviewed the Data Availability Statement and confirm that it accurately reflects the restricted-access nature of the registry data, in accordance with PLOS Medicine’s policy. The statement specifies the legal and institutional constraints and provides the appropriate URL for data access inquiries.

Figures and Tables:

* Please define all elements of box plots in the figure caption - center line, box limits and whiskers.

We have reviewed this point.

* Please provide titles and legends for all figures and tables (including those in Supporting Information files). Please define all acronyms used in each figure or table in its corresponding legend.

We have provided all required titles, legends, and acronym definitions for every figure and table, including those in the Supporting Information files.

* Please ensure that where relevant figures include 95% CIs.

We have reviewed this point.

* When a p value is given, please specify the statistical test used to determine it in the legend.

We have added descriptions of the statistical tests used to calculate each reported p value to all figures and tables where this information was previously not specified.

* In the Kaplan-Meier curve(s) please provide the number at risk for each time interval.

We confirm that all Kaplan–Meier curves now include the number at risk for each time interval.

* Where data points are discrete, please ensure that they are depicted in the figures as discrete data and not as a continuous line.

We have reviewed this point.

* Please provide the unadjusted comparisons as well as the adjusted comparisons in all relevant Tables

We have reviewed this point.

* Please specify the variables controlled for in all relevant Tables

We have reviewed this point.

* Please move Figure S14 and Table S2 into the main text.

We have moved Figure S14 and Table S2 into the main text as requested.

Line 55: Please clarify meaning of performance status

We have clarified this phrase by specifying that it refers to the patient’s functional performance status.

Line 61: Is the date range the date of diagnosis? Please clarify.

We have clarified that the date range refers to the year of HCC diagnosis.

Line 62: Please spell out acronyms

We have fully spelled out the acronyms.

Line 65: Please spell out BCLC

We have spelled out BCLC.

Line 70: Please add results using other LLMs (not only for ChatGPT)

We have added the results from the other LLMs as requested.

Please add results on BCLC-B to the Abstract

We have added the BCLC-B results to the Abstract in the revised manuscript.

Please clarify the version of ChatGPT (and of the other LLMs if relevant) and correct throughout.

We have clarified the versions of ChatGPT, Gemini, and Claude across the entire manuscript, and added consistent version labels to all figure and table legends, including those in the Supporting Information.

[Comments from the reviewers]

Reviewer #1: We thank the authors for addressing our previous comments. Some final remarks remain:

1. In the Impact of concordance between LLM-physician on OS section, it is stated that "In BCLC-B, survival was also better in matched patients, although the difference was relatively modest (HR, 1.091; P = 0.008; Fig 2C)". Would a HR > 1.0 indicate poorer survival instead?

Authors’ response) We thank the reviewer for pointing out this inconsistency. We agree that a hazard ratio > 1.0 indicates poorer survival. The sentence has been corrected in the revised manuscript to accurately reflect the direction of the association (page 14, line 274-275).

2. While it was stated in the reply to Point 5 that "If the observed effects were driven purely by a bias toward patients with better baseline prognosis, we would expect uniformly favorable OS in matched cases across all stages, which was not observed", such a bias would appear to imply more (and more-appropriate) matched cases for the BCLC-A stage (less-severe cases), and fewer (and less-appropriate) matched cases for the BCLC-C stage. It is thus unclear why there would be an expectation of uniformly favourable OS for matched cases across all stages, under the biased baseline prognosis assumption.

However, the details in S15 and S16 Table do suggest that when the LLM makes disagreements, the recommendations are generally similar in expected survival rate to the human assessment. This supports the authors' assertions from the clinical mechanism and within-treatment sensitivity analysis perspectives.

Nevertheless, it may remain more appropriate to analyze clinical utility of the LLM using an "[mean absolute] error in expected survival outcome" metric (which penalizes both under- and over-estimating the survival outcome), rather than a "lower/better expected survival outcome" metric. This is because as there is no counterfactual as to the actual impact of a changed treatment regime (as acknowledged in the response to Point 3 of the previous review), the actual observed survival might more appropriately be considered the ground truth in terms of prognosis correlation.

Authors’ response) We sincerely appreciate the reviewer’s insightful suggestion regarding a mean absolute error-based evaluation metric. We fully agree that mean absolute error is an important tool when assessing a model’s numerical survival predictions, as it symmetrically penalizes over- and under-estimation. However, mean absolute error is not applicable to the present study for several methodological reasons.

Our analysis did not involve asking LLMs to generate numerical estimates of expected survival. The models were tasked solely with recommending a treatment strategy based on structured clinical variables, not predicting survival durations or probabilities. Because no quantitative predicted-survival values exist, the mean absolute error-based evaluation cannot be meaningfully performed.

In addition, neither the registry dataset nor routine clinical workflows include clinician-generated numerical survival predictions. Thus, there is no real-world “ground truth prediction” against which an error metric could be defined. Any post-hoc numerical estimates generated solely for the purpose of computing mean absolute error would fall outside the study design and would not constitute validated or interpretable prognostic benchmarks.

More importantly, the goal of our study was not to evaluate predictive accuracy but to assess the associational clinical implications of treatment-selection concordance—namely, whether concordance or discordance with clinician decisions was associated with differences in observed survival within each BCLC stage. These observed outcomes were not used as a surrogate for “expected survival” or a prediction metric; rather, they reflect real-world trajectories under actual treatment pathways. Applying mean absolute error would require a fundamentally different study design focused on prognostic modeling, not treatment decision analysis.

Accordingly, we have clarified this distinction in the revised manuscript to prevent misinterpretation regarding the inapplicability of mean absolute error and the intended focus of our analyses (page 21, line 274-275).

Reviewer #2: Many thanks for addressing my comments. Just one point is outstanding:

- The Methods section should be explicit about the lack of a formal prompt engineering phase, and detail the background of the individuals that produced the standardised prompts--this is also part of the CHART checklist (point 5).

Authors’ response) We thank the reviewer for this important observation. We have revised the Methods section to explicitly state that no formal prompt-engineering phase was undertaken. We have also added details regarding the background of the individuals who developed the standardized prompts. Specifically, the revised manuscript now notes that all prompts were derived directly from AASLD/EASL guidelines and were drafted by two board-certified hepatologists and one clinical researcher with expertise in HCC (page 8, line 192-194).

Reviewer #3: None
